# Integrated genomic analyses of acral and mucosal melanomas nominate novel driver genes

**DOI:** 10.1186/s13073-022-01068-0

**Published:** 2022-06-16

**Authors:** Meng Wang, Ishani Banik, A. Hunter Shain, Iwei Yeh, Boris C. Bastian

**Affiliations:** 1 Department of Dermatology, University of California San Francisco, San Francisco, CA USA; 2 Helen Diller Comprehensive Cancer Center, University of California San Francisco, San Francisco, CA USA; 3 Department of Pathology, University of California San Francisco, San Francisco, CA USA

## Abstract

**Background:**

Acral and mucosal melanomas are aggressive subtypes of melanoma, which have a significantly lower burden of somatic mutations than cutaneous melanomas, but more frequent copy number variations, focused gene amplifications, and structural alterations. The landscapes of their genomic alterations remain to be fully characterized.

**Methods:**

We compiled sequencing data of 240 human acral and mucosal melanoma samples from 11 previously published studies and applied a uniform pipeline to call tumor cell content, ploidy, somatic and germline mutations, as well as CNVs, LOH, and SVs. We identified genes that are significantly mutated or recurrently affected by CNVs and implicated in oncogenesis. We further examined the difference in the frequency of recurrent pathogenic alterations between the two melanoma subtypes, correlation between pathogenic alterations, and their association with clinical features.

**Results:**

We nominated *PTPRJ*, mutated and homozygously deleted in 3.8% (9/240) and 0.8% (2/240) of samples, respectively, as a probable tumor suppressor gene, and *FER* and *SKP2*, amplified in 3.8% and 11.7% of samples, respectively, as probable oncogenes. We further identified a long tail of infrequent pathogenic alterations, involving genes such as *CIC* and *LZTR1*. Pathogenic germline mutations were observed on *MITF*, *PTEN*, *ATM*, and *PRKN*. We found *BRAF* V600E mutations in acral melanomas with fewer structural variations, suggesting that they are distinct and related to cutaneous melanomas. Amplifications of *PAK1* and *GAB2* were more commonly observed in acral melanomas, whereas *SF3B1* R625 codon mutations were unique to mucosal melanomas (12.9%). Amplifications at 11q13-14 were frequently accompanied by fusion to a region on chromosome 6q12, revealing a recurrent novel structural rearrangement whose role remains to be elucidated.

**Conclusions:**

Our meta-analysis expands the catalog of driver mutations in acral and mucosal melanomas, sheds new light on their pathogenesis and broadens the catalog of therapeutic targets for these difficult-to-treat cancers.

**Supplementary Information:**

The online version contains supplementary material available at 10.1186/s13073-022-01068-0.

## Background

Acral and mucosal melanomas account for approximately 3–4% and 1% of total melanoma cases in Caucasians [1–4], respectively, but are the dominant melanoma subtypes in non-Caucasian populations [5, 6]. Acral melanomas arise on the non-hair-bearing skin of palms, soles, and the nail apparatus, while mucosal melanomas primarily affect mucosal membranes of the nasopharynx and anogenital tracts. Contrary to cutaneous melanomas, which frequently originate from sun-exposed skin, the sites harboring acral and mucosal melanomas are relatively or completely shielded from environmental ultraviolet (UV) radiation. Both melanoma subtypes share similar genomic features such as low burdens of somatic mutations with an increased frequency of copy number and structural alterations instead [7–9]. The specific genetic alterations found in acral and mucosal melanomas only partially overlap with those in cutaneous melanomas [7]. For example, acral and mucosal melanomas have fewer *BRAF* mutations but more frequent *KIT* mutations, amplifications of *CCND1*, *CDK4*, and *MDM2*, and deletion of *SPRED1* [7, 8, 10–12].

Due to their comparatively lower incidence in western countries and distinctive features, acral and mucosal melanomas were excluded from The Cancer Genome Atlas (TCGA) [13], and the number of sequencing studies of these two subtypes have comparatively smaller sample sizes [8, 10, 11, 14–17]. Consequently, the power of discovery has been lower, and the landscape of somatic and germline driver events remains incompletely characterized. In this study, we compiled a dataset of human acral and mucosal melanomas from multiple previous studies [8, 10, 11, 13–16, 18–21] and performed a meta-analysis.

## Methods

### Data collection

We downloaded the mapped BAM files or, where available, unprocessed FASTQ sequencing files from whole-genome or whole-exome sequencing (WGS and WXS) of 154 acral and 93 mucosal melanomas from 11 previous studies [8, 10, 11, 13–16, 18–21] (Additional file 1: Table S1). Four patients had multiple tumor samples from the same cancer sequenced, and we included only the metastasis with the highest purity for analysis for these patients. We excluded 7 acral melanomas with target coverage under 25-fold or tumor cell content under 20%, leaving 147 acral and 93 mucosal melanomas, or 165 WGS and 75 WXS samples, for downstream analyses.

### Processing of sequencing data

For cases for which only BAM files were available [8, 10, 11, 13], we confirmed usage of hg19 and directly proceeded to downstream analyses. For the remaining samples, we mapped FASTQ data to the hg19 assembly, using the Burrows-Wheeler Aligner [22] and used Picard (http://broadinstitute.github.io/picard) to remove PCR duplicates and calculate insert size, followed by GATK [23] to perform base quality score recalibration and realign indel regions.

### Calling of somatic mutations

We used Mutect2 [24] and Strelka2 [25] to call somatic SNVs and short indels for each melanoma DNA, using its matched germline DNA as reference. Mutations identified by both tools were merged and annotated with Annovar [26]. We removed variants corresponding to single-nucleotide polymorphisms (SNPs) found in more than 1% of any of the populations in ExAC, GnomAD, 1000 Genome, dbSNP150, or NHLBI-ESP [27–31] to eliminate false positive somatic calls at loci with low germline coverage. For coding mutations flagged as “PASS” by both tools, a minimum of 4 reads were required to be included in further analyses. For mutations that passed only one tool, we required a coverage of 10 reads in tumor and normal samples, a minimum of 6 mutated reads in tumor and a maximum of one mutated read or 3% mutant allele frequency (MAF) in normal. We excluded indels flagged as “clustered_events” or “clustered_events;haplotype” by Mutect2. Noncoding mutations were required to be called as “PASS” by both tools, considering the abundant repetitive elements in noncoding regions [32]. To remove potential sequencing artifacts, we applied SOBDector [33] and removed SNVs that were labeled as “artifact.” For the final set of somatic mutations, we performed principal component analysis on the matrix of 96 classes of substitutions (Additional file 2: Fig. S1A and B), and observed moderate separation of WGS and WXS samples that was probably attributable to context differences between coding and noncoding regions. Several samples with distinct mutation signatures, including UV radiation, mismatch repair defect and temozolomide treatment, were scattered as outliers. Boxplots of median MAF for samples from several major studies were shown in Additional file 2: Fig. S1C.

### Identification of significantly mutated genes (SMGs)

We used 3 tools, dNdScv [34], MutPanning [35], and MutSigCV [36], to identify significantly mutated genes. For dNdScv, the R package was applied (https://github.com/im3sanger/dndscv). MutPanning was implemented through the GenePattern online server (https://cloud.genepattern.org/gp/pages/index.jsf). For MutSigCV, the GitHub version (https://github.com/getzlab/MutSig2CV) was used. Mutations located in exons or splice sites were extracted as input for the analysis.

### Calling germline mutations

We used HaplotypeCaller [37] to call germline mutations in the normal DNA. Mutations with fewer than 4 reads or an allele frequency under 25% were removed and the remaining mutations annotated using Annovar. We further removed common SNPs with > 1% mutation frequency in ExAC, GnomAD, 1000 Genome, dbSNP150, or NHLBI-ESP [27–31]. The pathogenicity of germline mutations was estimated using annotations from OncoKB [38] and ClinVar [39]. Mutations that were annotated as “benign/likely_benign” by Clinvar, as “neutral” or “inconclusive” by OncoKB, or were located very close to protein C-terminus were not considered as possible driver events. A pathogenic *JAK2* V617F mutation was identified in patient A_MELA_0061, with 45% allele frequency in the blood sample but 0 mutated reads (out of 62) in the corresponding tumor sample that retained both alleles of *JAK2*. The tumor and blood samples were confirmed to have originated from the same patient using the “CalculateContamination” function from GATK. This mutation was subsequently removed since it likely reflects clonal hematopoiesis [40] rather than a *bona fide* germline mutation.

### Determining the genetic ancestry of patients

To determine the genetic ancestry of included patients, we downloaded the phase 3 autosomal variants of the 1000 Genomes Project (ftp://ftp.1000genomes.ebi.ac.uk/vol1/ftp/release/20130502; PED file from: ftp://ftp.1000genomes.ebi.ac.uk/vol1/ftp/technical/working/20130606_sample_info/20130606_g1k.ped), and removed SNPs with minor allele frequency < 1%, multi-allelic variants, indels, as and those not located within exons or the 100-bp immediate flanking regions. For the remaining SNPs, we genotyped the corresponding loci in our normal samples using SAMtools and BCFtools [41], with a requirement of at least 10 reads. We further removed loci that failed genotyping in over half of the patients or had inconsistent alternative allele in any patient. We next applied PLINK v1.90b6.24 for downstream analyses. Specifically, data from our own cohort and the 1000 Genomes Project were converted to PLINK format and merged. From the merged dataset, we removed SNPs with allele frequency < 10% and further pruned to obtain the subset in approximate linkage equilibrium, with arguments “--maf 0.1 --indep 50 5 1.5”. The remaining SNPs were input for principal component analysis. Based on the closeness with 1000 genome populations, 96.7% (174/180) of patients with ancestry information available can be assigned to the correct ethnicity (Additional file 1: Table S1 and Additional file 2: Fig. S2).

### Identification of pathogenic mutations

We downloaded the list of 682 annotated cancer genes from OncoKB (as of April 14, 2021) [38] and included 21 additional genes of interest (Additional file 3: Table S2), which emerged during our analyses or had been implicated in melanoma or other cancer types. Germline and somatic mutations in any of these 703 genes were considered as pathogenic, if (1) annotated as “oncogenic,” “likely oncogenic,” or “hotspot” by the OncoKB database or (2) predicted to be damaging (nonsense, stoploss, frameshift or splice site) a known tumor suppressor gene.

### Mutational signature analysis of single-nucleotide substitutions

We used the R package SigProfilerExtractorR [42] (https://github.com/AlexandrovLab/SigProfilerExtractorR) with the matrix of 96 classes of substitutions in samples as input. The parameters “minimum_signatures” and “maximum_signatures” were set to be 3 and 15, respectively. The tool identified the most probable solution of de novo signatures, and subsequently decomposed them into combinations of COSMIC single base substitution (SBS) signatures (https://cancer.sanger.ac.uk/signatures/sbs/). These SBS signatures reflect different mutational processes. For instance, SBS7 is known as the “UV signature” because it is induced by exposure to UV radiation [43].

### Copy number analysis

We used FACETS to assess relative and absolute copy number variation (CNV), loss of heterozygosity (LOH), and tumor purity and ploidy [44]. Overall, our purity estimates appeared comparable or better than those from the original studies (Additional file 2: Fig. S3). For a few exceptions with less accurate FACETS estimates, we recalculated purities using either the median MAF of somatic mutations, or the median VAF of germline variants at hemizygous regions [45]. We then recalculated the absolute copy number (or *n*) of each segment using the below formula (*ρ* and *φ* represent tumor purity and ploidy, respectively) under the assumption of diploid genome (the log2 ratios of diploid segments were near 0).

1 $$\mathit{\log}2\ Ratio=\mathit{\log}2\left(\frac{\left(1-\rho \right)\ast 2+\rho \ast n}{\left(1-\rho \right)\ast 2+\rho \ast \varphi}\right)$$

To resolve visually suspicious regions of narrow homozygous deletions or amplifications that were not called by FACETS, we used the pre-segmented log2 ratios calculated by CNVkit [46], which was run separately for the WGS and WXS datasets to account for variations in coverage across studies. Amplifications were defined as copy number increases more than twofold than the estimated ploidy of the tumor and affecting a genomic region no larger than 10 megabases.

We applied GISTIC2 [47] to the segmented and purity-adjusted copy number data derived from FACETS to identify genomic regions with recurrent copy number alterations, likely harboring genes relevant in acral and mucosal melanoma. The copy number ratios were median centered by GISTIC2 prior to the analysis.

### Identifying structural variations (SV)

Delly2 [48] was used to identify somatic SVs in the WGS data.

### Estimation of microsatellite instability

MSIsensor [49] was used to estimate the level of microsatellite instability within coding regions in tumor DNAs compared to their matched germline reference DNA.

### Identifying driver events with distinct frequencies between the two melanoma subtypes and testing for the interdependence among driver events

We used Fisher’s exact test to compare the frequency of pathogenic alterations between the two subtypes and examine the correlations among different types of alterations. Altered tumor suppressor genes were defined as those with inactivating mutations or homozygous deletions. For oncogenes, amplification and pathogenic or likely pathogenic mutations were classified into separate groups. *BRAF* mutations were classified into class 1 (V600E and V600K) and other *BRAF* mutations. Overall, 35 groups of alterations, each observed in at least 10 samples, were included for analysis. For testing the interdependence of driver alterations, pairs of gene amplifications located on the same chromosomal arms were excluded.

### Identifying driver events associated with clinical features

For acral melanomas, the frequency of pathogenic alterations were compared between those originated from sole (*n* = 71) and subungual area (*n* = 37), while for mucosal melanomas, pairwise comparison was performed among those originated from the genitourinary (*n* = 20), gastrointestinal (*n* = 15), and sinonasal/oropharyngeal (*n* = 37) systems, respectively. We also identified 53 primary and 85 metastatic acral melanomas, and 33 primary and 43 metastatic/recurrent mucosal melanomas, respectively, and compared primary with metastatic/recurrent samples for the merged set of both melanoma subtypes, and separately for each subtype. We further compared mucosal melanomas from patients of European and Asian ancestries (*n* = 48 and 42, respectively). No such comparisons were performed for acral melanomas since most were of European ancestry (*n* = 131). Fisher’s exact test was used for the above comparisons. Survival analysis was performed using the R package “survival.”

## Results

### Description of the dataset

We compiled published sequencing data from 147 acral and 93 mucosal melanomas (Additional file 1: Table S1) and applied a uniform pipeline to call tumor cell content, ploidy, somatic and germline mutations, as well as CNVs, LOH, and SVs (Additional files 4: Table S3, Additional files 5: Table S4, and Additional files 6: Table S5). Acral and mucosal melanomas had low mutation burdens (median number of mutations per megabase 1.92, range = 0.48–80.13 and 2.24, range = 0.64–20.04, respectively. Additional file 2: Figs. S4 and S5), consistent with previous estimates and considerably lower than the mutation burden in cutaneous melanomas [8]. We note that a small subset of samples showed higher mutation burdens, with 11 acral and 2 mucosal melanomas having over 10 mutations per megabase, attributable to UV radiation (SBS7; *n* = 8), chemotherapy with temozolomide (SBS11) and/or platinum (SBS31) (*n* = 4), or DNA mismatch repair defects (SBS 21 and 26) due to homozygous deletion of *MLH1* in A_MELA_0271 (MSI score = 20.59) (Additional file 2: Figs. S4 and S5). Only one other melanoma, M_MELA_0581, showed evidence of mismatch repair deficiency (MSI score = 5.54. Additional file 2: Fig. S2), which was accompanied by biallelic inactivation of *MSH6*, but lacked an elevated mutation burden (2.24 per megabase) or mismatch repair deficiency-related signature, likely indicating that mismatch repair deficiency did not account for the majority of mutations in this melanoma.

### Identification of significantly mutated genes

**Fig. 1:**
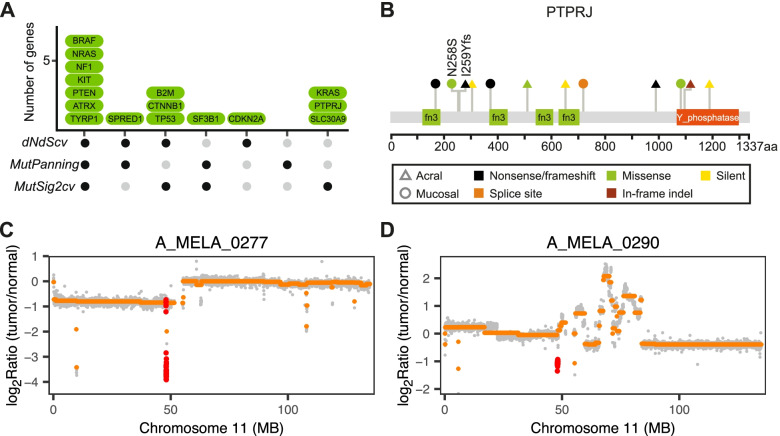
Recurring *PTPRJ* alterations in acral and mucosal melanomas. **A*** PTPRJ* is among the 16 SMGs identified by one or more of the three algorithms (*q* < 0.1) in the combined dataset of both subtypes. **B** Lollipop plot with somatic non-synonymous, splice site, and silent mutations in *PTPRJ*. **C**, **D** Focused homozygous deletions of *PTPRJ* in two acral melanomas

The algorithms dNdScv, MutPanning, and MutSigCV identified 16 significantly mutated genes (*q* < 0.1 by any tool) (Fig. 1A and Additional file 7: Table S6), 13 of which are well-documented driver genes in melanoma and known to activate the MAP kinase (MAPK) pathway (*BRAF*, *NRAS*, *NF1*, *KIT*, *SPRED1*, and *KRAS*) or other pathways (*PTEN*, *ATRX*, *CTNNB1*, *TP53*, *CDKN2A*, *SF3B1*, and *B2M*).

**Fig. 2:**
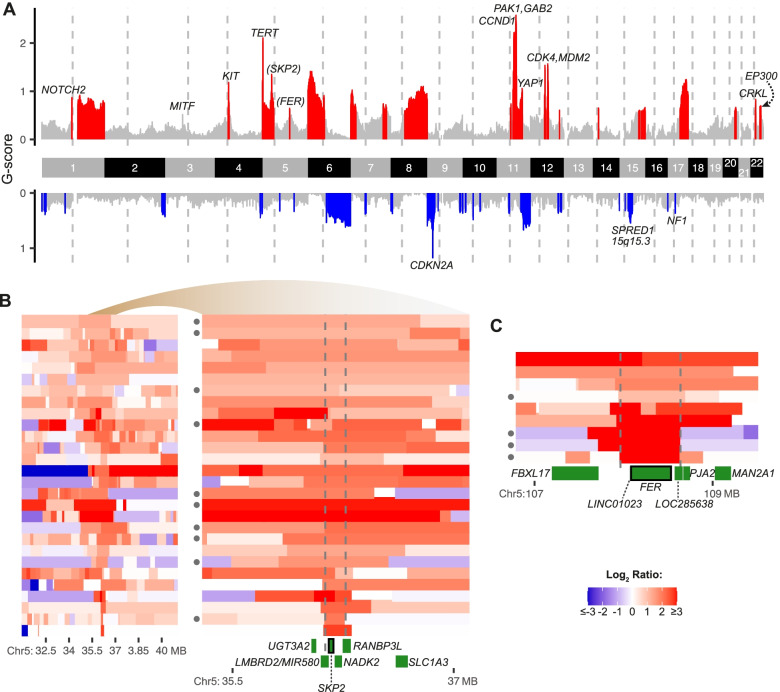
Candidate genes in genomic regions recurrently affected by focused amplifications or deletions. **A** GISTIC scores (*y*-axis) per chromosome (*x*-axis) for combined dataset of acral and mucosal melanomas. Recurrently (*q*-values < 0.1) amplified regions are colored red and recurrently deleted regions are colored blue, with relevant oncogenes and tumor suppressors labeled. Putative melanoma driver genes are marked in parentheses. **B**, **C** Minimally amplified regions (indicated in vertical lines) for amplifications of chromosome 5 with candidate genes. The filled circles on the left side indicate mucosal melanomas, with unlabeled cases representing acral melanomas. For panel **B**, the amplified region is shown at two different resolutions

Three additional significantly mutated genes were identified that are not yet firmly associated with melanoma. Mutations in the protein tyrosine phosphatase *PTPRJ*, 5 truncating, 3 missense, and 1 in-frame indel, were identified in 9 melanomas, 4 acral and 5 mucosal (Fig. 1B), and *PTPRJ* was homozygously deleted in two additional acral melanomas (Figs. 1C and 2D). Three and 1 of the truncating and missense mutations, respectively, were coupled with LOH. Together, 5 melanomas showed genetic evidence of biallelic inactivation, with either homozygous deletion or truncating mutation coupled with LOH. One recent study observed truncating *PTPRJ* mutations in 23% (7/31) of canine mucosal melanomas [50], while another study reported truncating mutations in both human (1/30) and canine (2/65) mucosal melanomas [51]. *PTPRJ* has been nominated as a tumor suppressor in other cancers [52]. These findings implicate *PTPRJ* likely as a tumor suppressor gene in acral and mucosal melanomas.

*TYRP1* was mutated in 10 melanomas (Additional file 2: Fig. S6A), 8 acral and 2 mucosal melanomas. Nine mutations (90%) were frameshifting deletions, in 8 of which 4 base pairs in codons 352–353 were removed. This recurrent deletion has been reported recently in acral melanoma [11], and an identical frameshift deletion has been identified as the cause of oculocutaneous albinism type 3 [53]. The mechanisms by which this alteration contributes to melanoma pathogenesis are not understood.

*SLC30A9* was mutated in 5 melanomas, 4 acral and 1 mucosal melanomas (Additional file 2: Fig. S6B). While 3 mutations were truncating, there was no evidence of biallelic inactivation in any case. *SLC30A9* has not been previously implicated in melanoma or other cancer types, and additional evidence is needed to support its role as a driver gene. Summary of all non-silent mutations of the three genes can be found in Additional file 8: Table S7.

When we performed the analysis separately for acral and mucosal melanomas (Additional file 2: Fig. S7A and B), only a single additional candidate gene was flagged as significantly mutated, *FYB1* in acral melanoma (Additional file 2: Fig. S7C). *FYB1* is involved in coagulation and T cell signaling and its role in cancer is unclear.

### Candidate cancer genes in recurrent yet previously uncharacterized amplicons

Acral and mucosal melanomas frequently have structural genomic arrangements and focal amplifications and deletions that target oncogenes and tumor suppressors, respectively [7, 8]. We applied GISTIC2 to the combined dataset and individually for each subtype to refine the boundaries of focal amplifications and deletions for genomic regions known to be recurrently altered in these melanoma subtypes (Fig. 2A, Additional file 2: Fig. S8) and to identify previously unappreciated sites affected by focal amplifications. Recurrent but previously little characterized amplifications were encountered at chromosome 5p13.2 (11.7% or 28/240 of cases; Fig. 2B) and at 5q21 (3.8%; Fig. 2C).

The amplicon at 5p13.2 (Fig. 2B) is frequently accompanied by other amplifications on chromosome 5p, including those targeting *TERT* at 5p15.33. It contains the *SKP2* oncogene, an E3 ubiquitin ligase that degrades the cyclin-dependent kinase inhibitor p27 and thereby facilitates S-phase entry [54]. SKP2 is frequently overexpressed in melanoma and other cancer types [10, 55]. *SKP2* amplification was not mutually exclusive with common alterations targeting genes that control S-phase entry such as *CDKN2A* or *CDK4* (*P* > 0.05). The minimally amplified region further includes *NADK2* and *MIR580*, genes without a direct connection to cancer and therefore less likely drivers.

The minimally amplified region at 5q21 (Fig. 2C) includes the non-transmembrane receptor tyrosine kinase *FER* as a candidate oncogene. Overexpression of FER has been identified in several cancer types [56, 57], and FER has been shown to activate MAP kinase signaling by phosphorylating receptor tyrosine kinases including EGFR and MET [58, 59]. *FER* amplifications were not mutually exclusive with other genetic alterations in this pathway. Of the 9 samples with *FER* amplification, 7 had genetic alterations of known MAPK drivers, including *NF1* inactivation in 5 samples (*P* = 0.013; Fisher’s exact test for co-occurrence between the 2 genes), and *KIT* and *SPRED1* alterations, each in 1 additional sample. FER is also involved in cell-cell adhesion through phosphorylating proteins such as CTTN and β-catenin [60] but *FER* amplification was not inversely correlated (*P* = 1) with *CTTN* amplification, a recurrent alteration in acral melanoma [61].

**Fig. 3:**
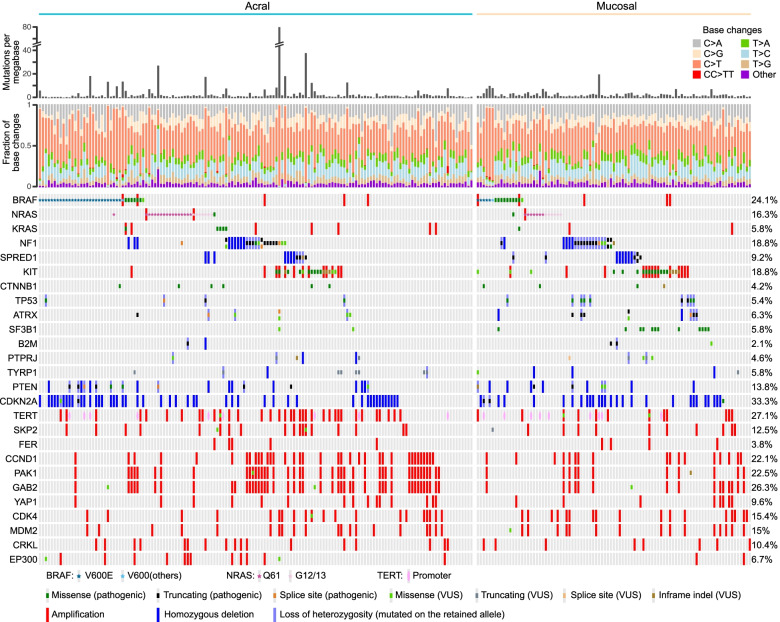
Summary of significantly altered cancer genes. Each column represents a case. For the tiling plot in the lower panel, genes are shown in rows and the type of alterations are detailed in the legend. Mutation burdens and the fraction of base changes were displayed in the upper panels

In summary, the above analyses revealed cancer genes (Fig. 3) that are significantly mutated or targeted by focused copy number alterations in acral and mucosal melanomas.

### Long tails of driver events in acral and mucosal melanomas

**Fig. 4:**
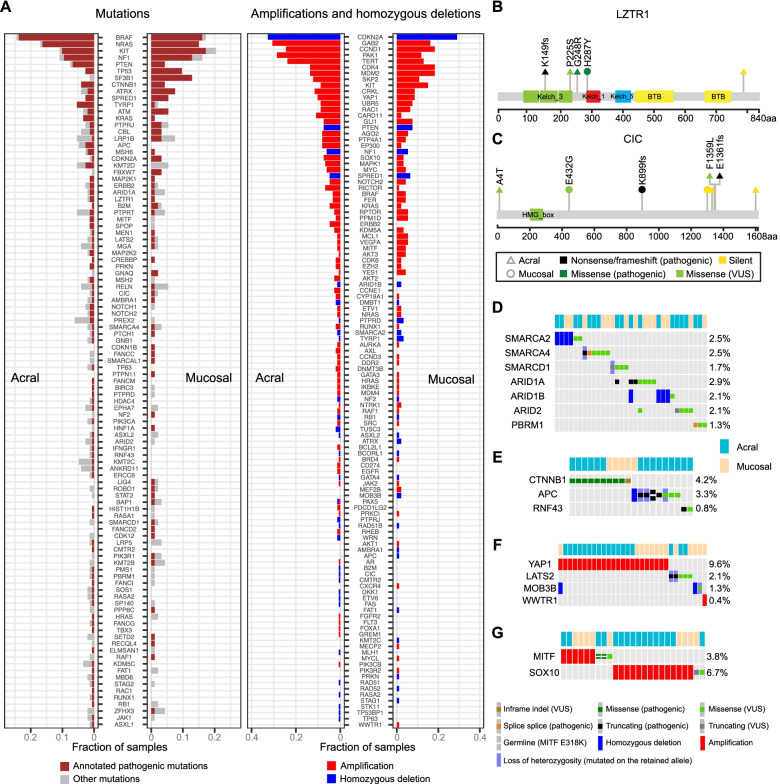
Long tails of driver events in acral and mucosal melanomas. **A** Genes with at least one pathogenic somatic or germline mutation (left panel), or amplifications and homozygous deletions (right panel). **B**, **C** Lollipop diagrams of mutations in *LZTR1* (**B**) and *CIC* (**C**). **D**–**G** Oncoprint plots of genes of SWI/SNF complex (**D**), WNT/β-catenin (**E**), Hippo pathway (**F**), and SOX-MITF (**G**) pathway with recurrent mutations

To identify additional putative driver events with low frequencies, we cross-referenced genetic alterations with OncoKB annotations (“Methods”). We observed that a considerable number of known cancer genes were altered by mutations, focal amplifications, and homozygous deletions, each in 5% or less of cases (Fig. 4A). These less common alterations affected multiple signaling pathways. In the MAP kinase pathway *MAPK1*, *RAC1*, *ERBB2*, *GNAQ*, *PTPN11*, *MAP2K1*, *MAP2K2*, *RAF1*, *SOS1*, *RASA1*, and *RASA2* alterations were observed (Additional file 2: Fig. S9). *NF1* homozygous deletion was observed in 5.8% (14/240) of tumors (Additional file 2: Fig. S10). Additional drivers that likely impact this pathway were *LZTR1* and *CIC*, which act as negative regulators of the MAPK pathway and each showed inactivating alterations in 3 tumors, with additional missense mutations of unknown significance in 1 and 3 other tumors, respectively (Fig. 4B, C, and Additional file 2: Fig. S9). LZTR1 functions as a component of the CUL3 E3-ligase complex, which mediates the degradation of RAS and is a tumor suppressor gene that can cause schwannomatosis and Noonan syndrome [62, 63]. CIC is a transcriptional repressor of ETS transcription factors and other genes downstream of the MAPK pathway and is a recurrently inactivated tumor suppressor gene in gliomas and other cancer types [64, 65].

Members of the SWI/SNF complex were altered in 13.8% (33/240) of samples (Fig. 4D), with *SMARCA2*, *SMARCA4*, *SMARCD1*, *ARID1A*, *ARID1B*, *ARID2*, or *PBRM1* each altered in approximately 2% of cases. Interestingly, *SMARCA2* and *ARID1B* alterations were mainly homozygous deletions (Additional file 2: Fig. S11), possibly because they map to chromosomes 9p and 6q, respectively, which are commonly affected by arm-level losses in melanoma, whereas alterations of other genes constituted indels and SNVs.

*CTNNB1*, *APC*, and *RNF43* of the WNT/β-catenin pathway were altered in 4%, 3%, and 0.8% samples, respectively (Fig. 4E).

Genes in the Hippo pathway were altered in 13.8% (33/240) of samples (Fig. 4F), most commonly by *YAP1* amplification (9.6%). Inactivating mutations and mutations with unknown significance of *LATS2* and *MOB3B* were observed in 2.1% and 1.3% of cases. *WWTR1* was altered in 1.3% by amplification (*n* = 1) and mutations with unknown significance (*n* = 2).

*SOX10* and *MITF*, genes involved in melanocyte development and differentiation, were amplified (5.8% and 2.5%, respectively), or mutated (1.5% and 0.8%) in 10.4% (25/240) of cases (Fig. 4G).

### Germline mutations

We identified 52 possible pathogenic germline mutations affecting 37 genes in 44 patients (18.3%) (Additional file 2: Fig. S12). *MITF* E318K, a known melanoma predisposing mutation [66, 67], was observed in 2 patients. The remainders were heterogeneous loss-of-function mutations of tumor suppressor genes, mostly operative in the DNA damage response and repair pathways. Only in 9 patients were the germline mutations accompanied with loss of the wild-type allele in the tumor sample, providing evidence that the germline alterations were functionally relevant for the development of the melanoma (Additional file 2: Fig. S12). Genes with such combined biallelic inactivation were *ATM* (2 patients), *PRKN* (2 patients), *PTEN*, *PTCH1*, *CMTR2*, *FANCG*, and *SMARCAL1* (1 patient each). For the remaining 41 germline mutations, the melanoma retained the corresponding wild-type allele.

### Differences in the genetic alterations of acral and mucosal melanomas

**Fig. 5:**
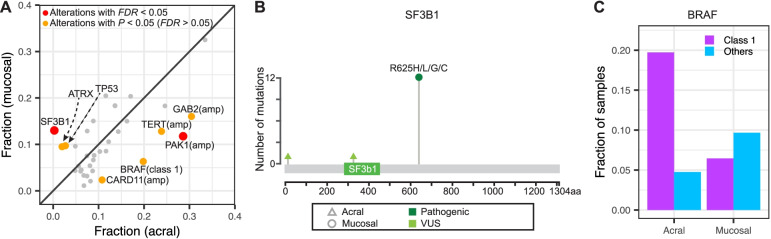
Differences of genetic alterations between acral and mucosal melanomas. **A** Scatter plot of mutation frequencies for the two subtypes. **B** Lollipop diagram of *SF3B1* mutations that were exclusive to mucosal melanomas. **C*** BRAF* class 1 (V600 codon) mutations were common in acral melanoma (*FDR* = 0.053), with no differences for other *BRAF* mutations (*FDR* = 0.46)

We next compared the frequency of the 35 recurrent alterations, defined as at least 10 occurrences, between acral and mucosal melanomas. Inactivating mutations and homozygous deletions of a tumor suppressor gene were considered jointly as inactivation, whereas amplification and pathogenic mutations of the same oncogene were considered individually. *BRAF* mutations were classified as class 1 (V600E: *n* = 33; and V600K: *n* = 2) or other *BRAF* mutations (*n* = 17). Two alterations were distributed differently among the two melanoma subtypes after accounting for multiple testing (Fig. 5A). *SF3B1*^R625^ mutations were present in 12.9% of mucosal melanomas but were absent in acral melanoma (*FDR-*corrected *P*-value, Fisher’s exact test = 0.00026) (Fig. 5B). *PAK1* amplifications were more frequent in acral melanoma (28.6% vs. 11.8%; *FDR* = 0.041). Acral melanomas also had more frequent *BRAF* class 1 mutations (Fig. 5C; 19.7% vs. 6.5%), and amplifications of *GAB2* (30.6% vs. 16.1%), *TERT* (23.8% vs. 12.9%), *CARD11* (12.2% vs. 3.2%), and *ERBB2* (4.8% vs. 0%), whereas mucosal melanomas had more frequent *ATRX* (9.7% vs. 2%) and *TP53* (9.7% vs. 2.7%) alterations; however, these differences did not exceed significance threshold after correction for multiple testing.

GISTIC2 comparison nominated amplifications at 22q13.2 (containing *EP300*), which was exclusively present in acral melanoma (*q* = 0.0016) and 3p13 (containing *MITF*), exclusive to mucosal melanoma (*q* = 0.22), but these differences did not reach formal levels of significance (*P* > 0.05).

### Genetic interactions among driver events

**Fig. 6:**
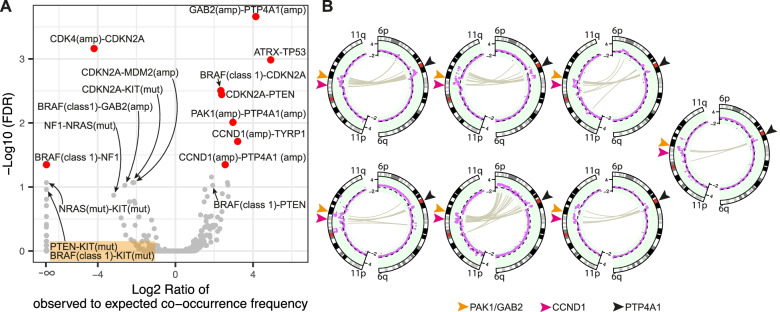
Interactions between genetic alterations. **A** Volcano plot with dots representing pairs of two separate genetic alterations. The log_2_ ratio of the observed and expected co-occurrence frequency is plotted on the *x*-axis against the −log_10_ (*FDR*) on the *y*-axis. Pairs with *FDR* (corrected *p*-value from Fisher’s exact test) < 0.05 are colored in red. **B** Circos plots showing clusters of translocations (gray lines) joining regions on chromosome 6q12 and 11q13-14 in 7 samples. The outer track shows the chromosome ideograms; the middle track shows the log_2_ ratio of tumor to normal copy number (purple dots) with the dashed black line indicating 0

We observed the following correlations among the 35 recurring genetic alterations (Fig. 6A). *ATRX* inactivation correlated with *TP53* mutations (*FDR* = 0.001), consistent with the notion that these two alterations cooperate in facilitating the alternative lengthening of telomeres [68]. This co-occurrence of *ATRX* and *TP53* alterations was only evident in mucosal melanoma (Additional file 2: Fig. S13). While either *ATRX* or *TP53* were altered in 7 acral melanomas, none harbored alteration of both genes. *BRAF* class 1 mutations, *PTEN* and *CDKN2A* inactivation, co-existed frequently, consistent with reports that *BRAF* V600E cooperates with *PTEN* and *CDKN2A* alterations [69, 70]. Amplification of chromosome 11q13-14, including the candidate genes *CCND1*, *PAK1*, and *GAB2*, frequently co-occurred with amplifications of chromosome 6q12. The WGS data revealed recurrent structural alterations joining sequences from both regions (Fig. 6B). We observed clusters of translocations between 6q12 and 11q13-14 in 46.7% (7/15) of samples that had amplifications of both regions. Thus, the co-amplification between 6q12 and 11q13-14 is likely partially driven by recurrent structural alterations between the two regions rather than functional cooperation of genes contained within these regions. The 6q12 locus harbors *PTP4A1*, a protein tyrosine phosphatase, previously implicated in cancer [71]. *TYRP1* alterations also correlated with *CCND1* amplification.

The following genetic alterations were inversely correlated (Fig. 6A). *CDK4* amplification and *CDKN2A* inactivation were mutually exclusive (*FDR* = 0.00069), consistent with prior reports [7]. *KIT* mutations were mutually exclusive with *PTEN* inactivation (*FDR* = 0.1), *CDKN2A* (*FDR* = 0.085) alterations, and *BRAF* class 1 mutations (*FDR* = 0.1). Samples with *CDKN2A* alterations also had fewer *MDM2* amplifications, but this may be due to the frequent co-amplification of *MDM2* and *CDK4*, which both reside on chromosome 12q, and driven by the anti-correlation of *CDK4* amplification and *CDKN2A* inactivation. Several mutations in the MAPK pathway showed a pattern of mutual exclusivity, but only *BRAF* class 1 mutation and *NF1* alterations reached the cutoff of *FDR* < 0.05, possibly reflecting the limited statistical power of our study. Melanomas with *BRAF* class 1 mutations harbored fewer amplifications of *GAB2* (*FDR* = 0.099), *CCND1*, and *PAK1* (both *FDR* = 0.18). However, we observed significantly fewer structural variations overall in melanomas with *BRAF* class 1 mutations compared to those without (Additional file 2: Fig. S14; 136 vs. 234 junctions on average; *P* = 0.0015), which remains significant after accounting for tumor purity and ploidy. As previously suggested, this may indicate that the *BRAF* V600E mutated melanomas on acral skin are likely different and more akin to cutaneous melanomas on skin with low cumulative sun-induced damage [12].

### The association of genetic alterations with clinical features

**Fig. 7:**
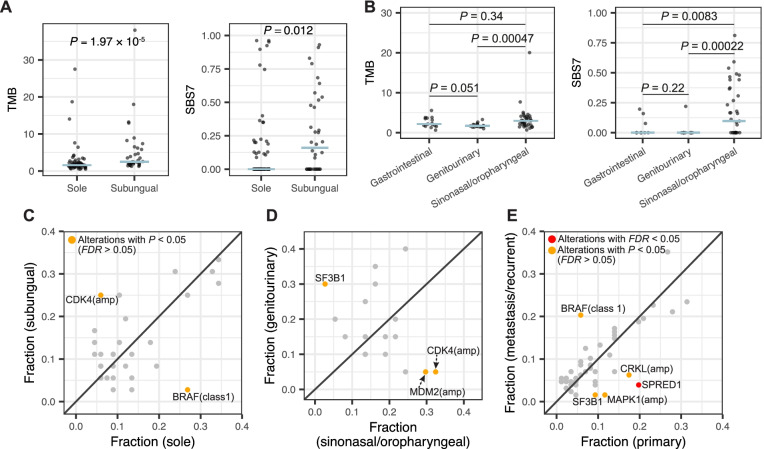
The association of genetic alterations with clinical features. **A**, **B** The comparison of tumor mutation burden and fraction of SBS7 mutations between sole and subungual acral melanomas (**A**), and across mucosal melanomas from different subsites (**B**). The blue segments denote the medians. **C**–**E** Scatter plots of mutation frequencies between sole and subungual acral melanomas (**C**), between sinonasal / oropharyngeal and genitourinary mucosal melanomas (**D**), and between primary and metastatic samples for the merged set of both melanoma subtypes (**E**)

Subungual melanomas (*n* = 37) showed significantly higher mutation burden (Fig. 7A; median mutations per megabase = 2.48 vs 1.6; *P* = 1.97 × 10^−05^ from Wilcoxon rank-sum test) and fraction of mutations that are attributable to UV signature SBS7 (*P* = 0.012), compared to melanomas originated from sole (*n* = 71). *BRAF* class 1 mutations occurred predominately in sole (Fig. 7C; 25.4% vs. 2.7%) compared to subungual melanoma, in contrast with *CDK4* amplification (5.6% vs. 24.3%). For mucosal melanomas, significantly higher mutation burden was observed in sinonasal/oropharyngeal (*n* = 37) compared to genitourinary melanomas (*n* = 20) (Fig. 7B; median mutations per megabase = 2.98 vs. 1.71). Sinonasal/oropharyngeal melanomas also showed significantly higher fraction of SBS7 mutations compared to the other mucosal melanoma groups (*P* < 0.01), which is consistent with a recent study [10]. *CDK4* (Fig. 7D; 32.4% vs. 5%) and *MDM2* (29.7% vs. 5%) amplifications were more frequently altered in sinonasal/oropharyngeal compared to genitourinary melanomas, in contrast with *SF3B1* mutations (2.7% vs. 30%) between the two subsites. We also found that *BRAF* class 1 mutations (20.3% vs. 5.8%) were more frequent in metastatic melanomas, while *SPRED1* inactivation (3.9% vs. 19.8%), *SF3B1* mutations (1.6% vs. 9.3%), and amplifications of *CRKL* (6.3% vs. 17.4%) and *MAPK1* (1.6% vs. 11.6%) were more frequent in primary samples (Fig. 7E and Additional file 2: Fig. S15). Only *SPRED1* inactivation remained significant after correction for multiple testing. We further compared mucosal melanoma from patients of European and Asian ancestry (*n* = 48 and 42; Additional file 2: Fig. S15E) and found that *SF3B1* mutations were predominately observed in the former (22.9% vs. 2.4%), while *SKP2* amplifications were more frequent in the latter (4.2% vs. 19%). However, these associations were no longer significant after correction for multiple testing.

When combining both melanoma subtypes, we found that *KIT* (Additional file 2: Fig. S15F; *P* = 0.026 from log-rank test) and *TP53* (*P* = 0.043) mutations were associated with poorer survival, while patients with *CCND1* amplification had better outcome (*P* = 0.027). For mucosal melanomas, *PTEN* altered patients (*n* = 8) had poorer survival (Additional file 2: Fig. S15G; *P* = 2.33 × 10^−05^). Considering the limited sample size and strong variation in patient age, tumor stage and treatment condition, further study is probably needed to validate these.

## Discussion

**Fig. 8:**
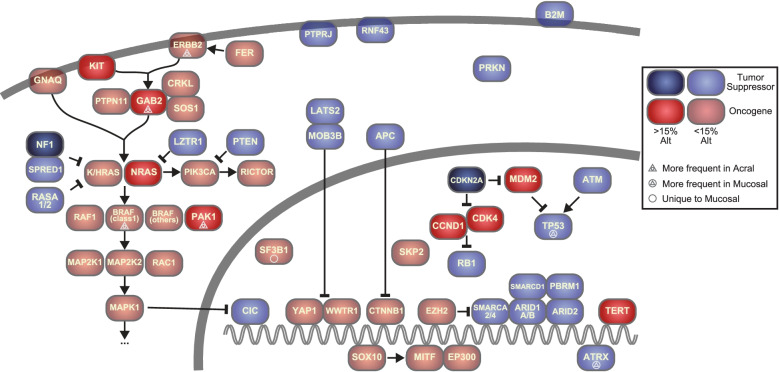
Major signaling pathways mutated in acral and mucosal melanoma

By integrating and analyzing samples from multiple previous studies, we present a wider view of the genetic landscapes of acral and mucosal melanomas (Fig. 8), provide additional support for the pathogenetic relevance of previously nominated genes, and implicate additional likely driver genes such as *PTPRJ*, *FER*, and *SKP2* altered in a subset of acral and mucosal melanomas.

PTPRJ is a member of the transmembrane protein tyrosine phosphatase family. PTPRJ promotes cell adhesion and inhibits PDGFR signaling [72], and its loss may promote meningioma progression through interacting with *NF2* loss [73]. The receptor tyrosine kinase FER activates MAP kinase pathway signaling and regulates cell adhesion in cancer cells [58, 59]. In our dataset, melanomas with *FER* amplification had frequent *NF1* inactivation but lacked *BRAF* and *NRAS* alterations. The findings indicate a possible role for *FER* amplification in driving the MAPK pathway, similar to KIT, another receptor tyrosine kinase that can be amplified or mutated in acral and mucosal melanoma, frequently also in conjunction with *NF1* mutations [74]. Functional studies have shown that inhibition of *FER* slows the proliferation and motility of melanoma cell lines [75]. *SKP2* is involved in G1/S cell cycle transition by degrading the cyclin-dependent kinase inhibitor 1B (CDKN1B, also referred to as p27 or KIP1) [54]. It is recurrently amplified in non-small cell lung cancer. There was no inverse correlation with other common genetic alterations of the G1/S checkpoint, indicating that *SKP2* amplification may not be redundant with *CDKN2A* inactivation. Overexpression of SKP2 has indicated worse survival in some studies [76]. Pharmacological inhibitors for FER [77] and SKP2 [78] are available, and further investigation is needed to study their roles in acral and mucosal melanomas and determine whether they constitute therapeutic targets.

Our data reveals a long list of low-frequency driver genes, which is likely to expand as more samples are being sequenced, following the experience in cutaneous melanoma [79, 80] and other cancers [81]. Among the 198 genes that had at least one pathogenic alteration, 83.8% (166/198) were present in fewer than 5% of cases, and 38.4% (76/198) were observed only once. These infrequently altered genes and pathways likely reflect the diverse trajectories that acral and mucosal melanocytes can take when evolving into melanomas and indicate that more alterations are to be discovered in these cancers.

We found germline mutations predicted to impact gene function in 18% of acral and mucosal melanoma patients, mainly functioning in the DNA damage sensing and repair pathways, similar to what has been seen in other cancer types [82]. In our analysis, only a small fraction of these germline alterations were accompanied by losses of the remaining allele in the tumors that would be required for complete inactivation. This suggests that the majority of these germline events may not contribute to tumor initiation and progression. The presence of the *MITF* E318K mutation in the germline of acral melanoma patients indicates that this mutation predisposes not only to cutaneous melanoma as has been previously reported [66], but may also predispose to acral melanoma. Also, none of the two *MITF* E318K cases contained *BRAF* V600 mutation. Mutations of *PRKN* are associated with hereditary Parkinson’s disease. The parkin protein is a component of a E3-ubiquitin ligase complex and involved in mitochondrium homeostasis and apoptosis. Recurrent loss-of-function mutations of *PRKN* occur in several cancer types where it acts as a tumor suppressor gene [83, 84]. Our finding of recurrent germline and somatic inactivating alterations suggests that *PRKN* probably also functions as a tumor suppressor in acral melanoma.

While acral and mucosal melanomas share many features such as their origin from sun-shielded or -protected sites, a precursor stage with a lentiginous growth pattern, and a high degree of chromosomal instability with frequent gene amplifications and structural rearrangements, our meta-analysis also adds to the emerging genetic differences between the two subtypes. Concordant with prior reports, pathogenic *SF3B1* mutations were found only in mucosal melanoma. SF3B1 is involved in RNA splicing, and the hotspot mutation recurrent in mucosal melanoma changes the splicing of several cancer genes, including the non-canonical BAF complex member BRD9 [85]. Interestingly, similar *SF3B1* mutations are found in a subset of uveal melanomas, another non-UV related melanoma subtype, which is genetically characterized by constitutive activation of the Gαq signaling pathway [86, 87]. Activating mutations of the Gαq pathway were also reported to occur in some mucosal melanomas [88] but were infrequent in our meta-analysis. The concurrent mutations of *ATRX* and *TP53* in mucosal melanomas suggest that alternative lengthening of telomere pathway is operative in some mucosal melanomas. *BRAF* class 1 mutations and 11q13-14 amplifications were more common in acral melanoma and tended to be mutually exclusive of each other. In summary, the subtle but reproducible differences in mutation patterns between acral and mucosal melanoma may reflect variations among their cells of origin or their micro-environments, selecting for different alterations.

Acral melanomas with *BRAF* class 1 mutations, especially V600E, had fewer structural variations and focal amplifications [12], than those without and thus resemble cutaneous melanomas. This raises the possibility that melanomas on acral are of two different types, with a *bona fide* and unique acral type with an admixture of cutaneous melanoma of the World health Organization (WHO) low-CSD subtype, i.e., melanomas on the sun-exposed skin with low degree of cumulative sun damage [89]. A recent study on acral nevi identified *BRAF* V600E mutation in 86% of samples [90], which is similar to what was observed in cutaneous nevi that considered the precursors of low-CSD melanomas [45]. Another study identified transcriptome-level differences between acral and cutaneous melanocytes [91]. Further investigation is needed to determine whether acral sites contain different sublineages of melanocytes. Acral skin has a unique reservoir of melanocyte stem cells in the sweat glands, abundant in acral skin [92], where as in most of the body’s skin melanocyte stem cells are associated with hair follicles [93].

While our study expands the view of the genetic landscapes of acral and mucosal melanoma, the number of tumors sequenced is still comparatively low and additional driver mutations likely remain to be discovered. An estimated 300 tumors would be needed to identify SMGs with 5% mutation frequency or higher, based on the average mutation burden of the two subtypes [36]. To increase the sample size, we merged both acral and mucosal melanomas for most of the analyses. This helped identify driver genes that are shared by both subtypes and does not permit exhaustive characterization of driver mutations unique to either subtype. The sample size also limited the comparison of mutation patterns and interactions between genetic alterations.

## Conclusions

In summary, our meta-analyses of 147 acral and 93 mucosal melanomas identified *PTPRJ*, *FER*, *SKP2*, *LZTR1*, *CIC*, and *PRKN* as part of a long tail of driver events that deserves further study and characterization. While the two melanoma subtypes have many features in common, the reproducible genetic differences support the notion that acral and mucosal melanoma are biologically distinct entities.

## Supplementary Information

**Additional file 1: Table S1.** A summary of all samples that were included in this study.

**Additional file 2: Figure S1.** Principal component analyses of the matrix of 96 classes of substitutions and boxplots of the mutant allele frequencies (MAFs) for samples from 6 studies. **Figure S2.** Principal component analysis showing the ethnicities of patients. **Figure S3.** Tumor purities estimated through FACETS were comparable with those from the original studies with few exceptions (red dots). For the Newell et al studies we observed larger discrepancies between our and the original estimates for hyperdiploid samples compared to near diploid samples, indicating that the original estimates probably did not account for tumor ploidy. **Figure S4.** Features of somatic mutations in acral melanomas. **Figure S5.** Features of somatic mutations in mucosal melanomas. **Figure S6.** Lollipop plots showing somatic non-synonymous mutations on *TYRP1* and *SLC30A9*. **Figure S7.** Significantly mutated genes (SMGs) in acral and mucosal melanomas. **Figure S8.** The GISTIC scores calculated from acral melanoma and mucosal melanoma samples. **Figure S9.** Oncoprint plots showing the alterations of genes that are involved in the MAPK signaling pathway. **Figure S10.** Copy number profiles showing homozygous deletion of *NF1*. **Figure S11.** Copy number profiles showing homozygous deletion of *SMARCA2* and *ARID1B*. **Figure S12.** Tiling plot of germline mutations. **Figure S13.** The mutual exclusivity and co-occurrence between pairs of driver events in acral melanoma and mucosal melanoma. **Figure S14.** Fewer structural variation (SV) junctions for acral and mucosal melanomas with *BRAF* class 1 mutations compared to others. **Figure S15.** The association of genetic alterations with clinical features.

**Additional file 3: Table S2.** The list of 703 cancer genes.

**Additional file 4: Table S3.** The list of somatic mutations in exons and splice sites for all samples.

**Additional file 5: Table S4.** The copy number data for all samples.

**Additional file 6: Table S5.** The output of Delly2 for WGS samples.

**Additional file 7: Table S6.** The outputs of dNdScv, MutPanning and MutSigCV.

**Additional file 8: Table S7.** All non-silent mutations from *PTPRJ*, *TYRP1* and *SLC30A9*.

## Data Availability

Sequencing data were downloaded from European Genome-phenome (EGA) archive under accession numbers EGAD00001003388 (https://ega-archive.org/datasets/EGAD00001003388) [8], EGAD00001004409 (https://ega-archive.org/datasets/EGAD00001004409) [10], EGAD00001005500 (https://ega-archive.org/datasets/EGAD00001005500) [11], EGAD00001000944 (https://ega-archive.org/datasets/EGAD00001000944) [15] and EGAD00001000945 (https://ega-archive.org/datasets/EGAD00001000945) [16], and from Database of Genotypes and Phenotypes (dbGaP) under accession numbers phs000178 (https://www.ncbi.nlm.nih.gov/projects/gap/cgi-bin/study.cgi?study_id=phs000178.v11.p8) [13], phs001036 (https://www.ncbi.nlm.nih.gov/projects/gap/cgi-bin/study.cgi?study_id=phs001036.v1.p1) [14], and phs000452 (https://www.ncbi.nlm.nih.gov/projects/gap/cgi-bin/study.cgi?study_id=phs000452.v3.p1) [18–21]. Details about the samples can also be found in Additional file 1: Table S1. The codes used for data analysis in the article are available at https://github.com/Bioinfowangm/ac_mu_analysis [94].

## Abbreviations

UV: Ultraviolet
TCGA: The Cancer Genome Atlas
WGS: Whole-genome sequencing
WXS: Whole-exome sequencing
SNP: Single-nucleotide polymorphism
MAF: Mutant allele frequency
SMG: Significantly mutated gene
SV: Structural variation
CNV: Copy number variation
LOH: Loss of heterozygosity
SBS: Single base substitution
MSI: Microsatellite instability
MAPK: Mitogen-activated protein kinase
WHO: World Health Organization
CSD: Cumulative sun damage
