# Supplementary material for: Integrated genomic analyses of acral and mucosal melanomas nominate novel driver genes
Source: Genome Med. 2022 Jun 16;14:65. doi: 10.1186/s13073-022-01068-0 (PMC9202124; doi:10.1186/s13073-022-01068-0)
Supplement: Supplementary file 2 — Additional file 2: Figure S1. Principal component analyses of the matrix of 96 classes of substitutions and boxplots of the mutant allele frequencies (MAFs) for samples from 6 studies. Figure S2. Principal component analysis showing the ethnicities of patients. Figure S3. Tumor purities estimated through FACETS were comparable with those from the original studies with few exceptions (red dots). For the Newell et al studies we observed larger discrepancies between our and the original estimates for hyperdiploid samples compared to near diploid samples, indicating that the original estimates probably did not account for tumor ploidy. Figure S4. Features of somatic mutations in acral melanomas. Figure S5. Features of somatic mutations in mucosal melanomas. Figure S6. Lollipop plots showing somatic non-synonymous mutations on TYRP1 and SLC30A9. Figure S7. Significantly mutated genes (SMGs) in acral and mucosal melanomas. Figure S8. The GISTIC scores calculated from acral melanoma and mucosal melanoma samples. Figure S9. Oncoprint plots showing the alterations of genes that are involved in the MAPK signaling pathway. Figure S10. Copy number profiles showing homozygous deletion of NF1. Figure S11. Copy number profiles showing homozygous deletion of SMARCA2 and ARID1B. Figure S12. Tiling plot of germline mutations. Figure S13. The mutual exclusivity and co-occurrence between pairs of driver events in acral melanoma and mucosal melanoma. Figure S14. Fewer structural variation (SV) junctions for acral and mucosal melanomas with BRAF class 1 mutations compared to others. Figure S15. The association of genetic alterations with clinical features. [file 13073_2022_1068_MOESM2_ESM.pdf]

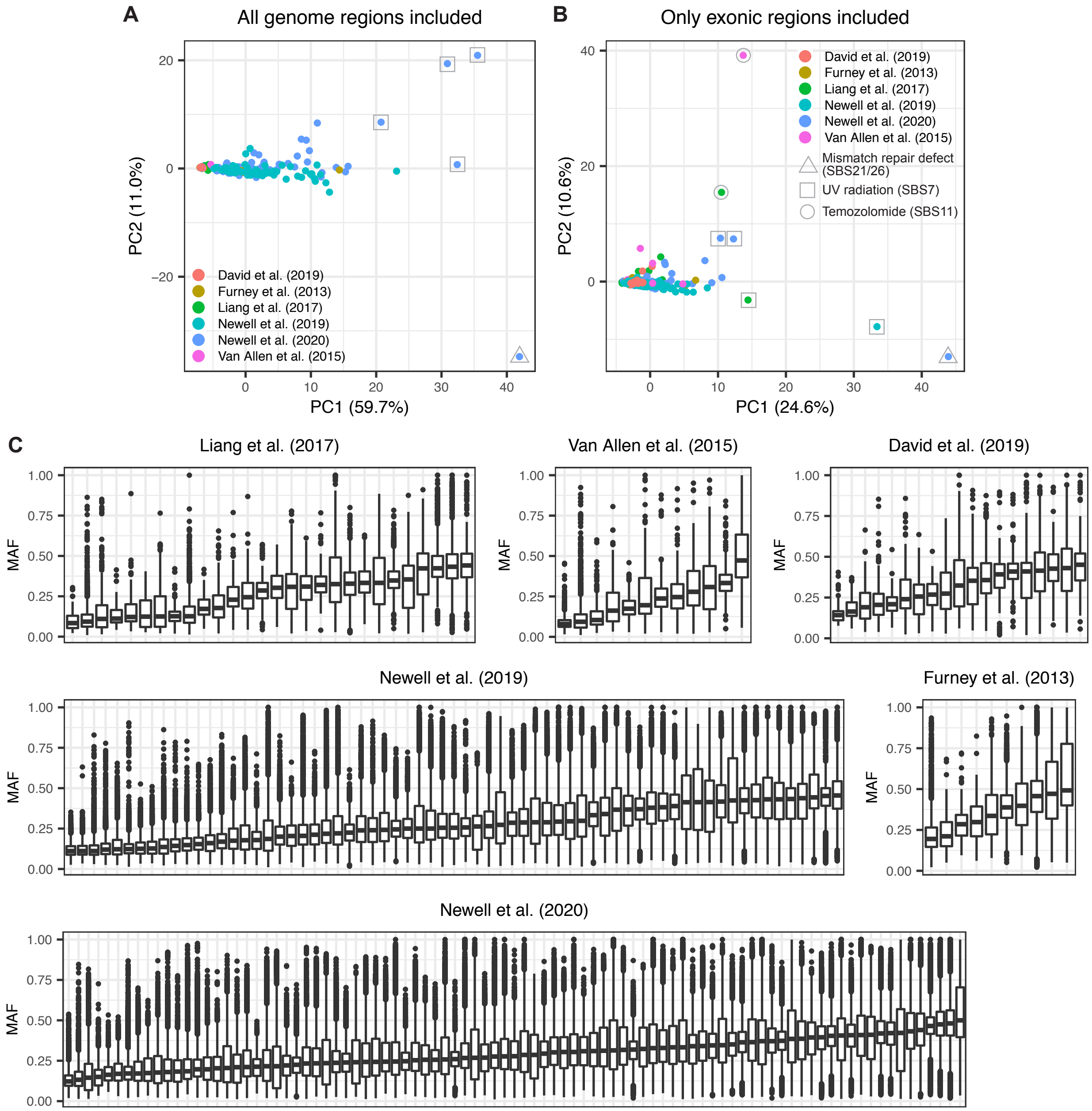

Figure S1. Principal component analyses of the matrix of 96 classes of substitutions and boxplots of the mutant allele frequencies (MAFs) for samples from 6 studies.

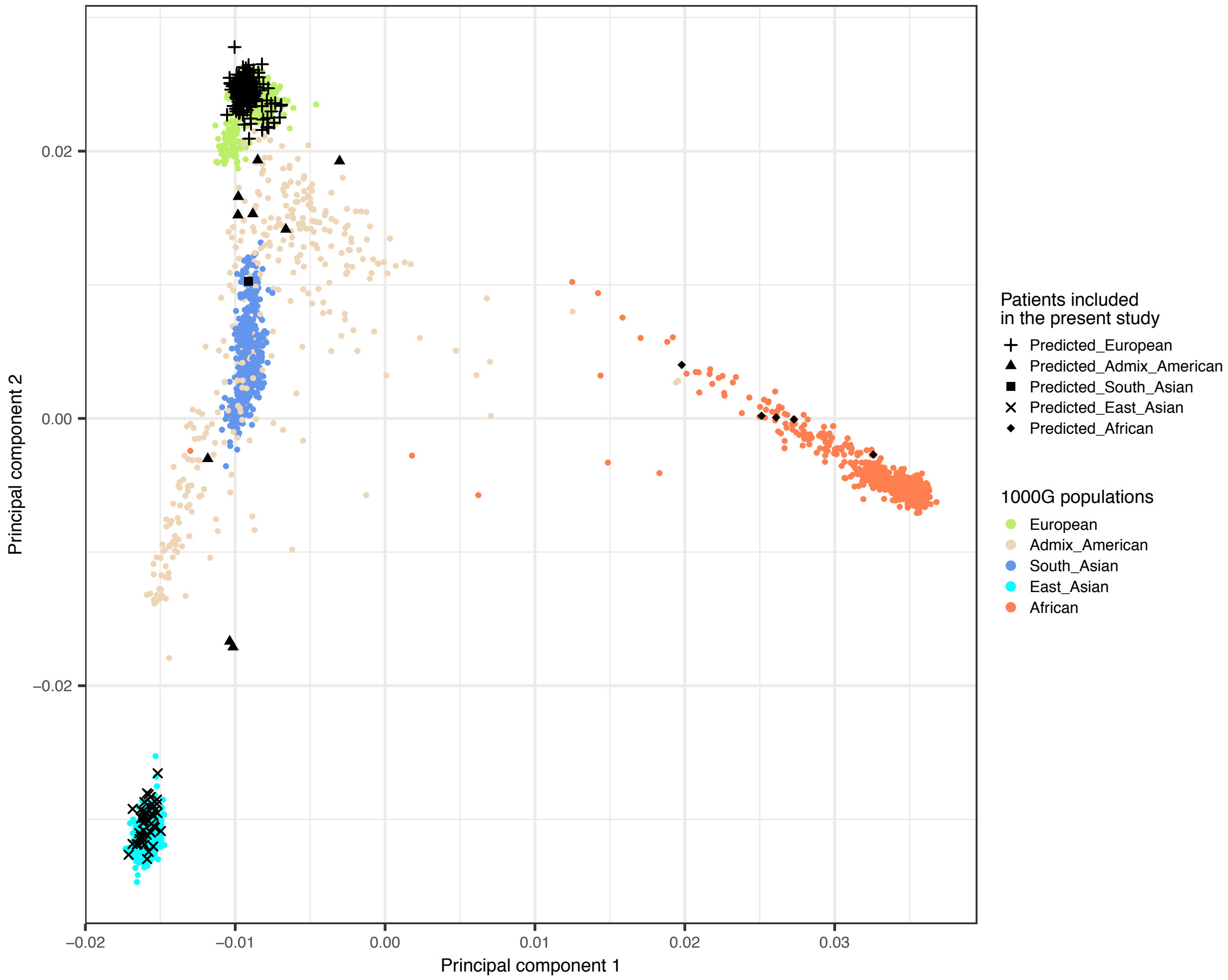

Figure S2. Principal component analysis showing the ethnicities of patients.

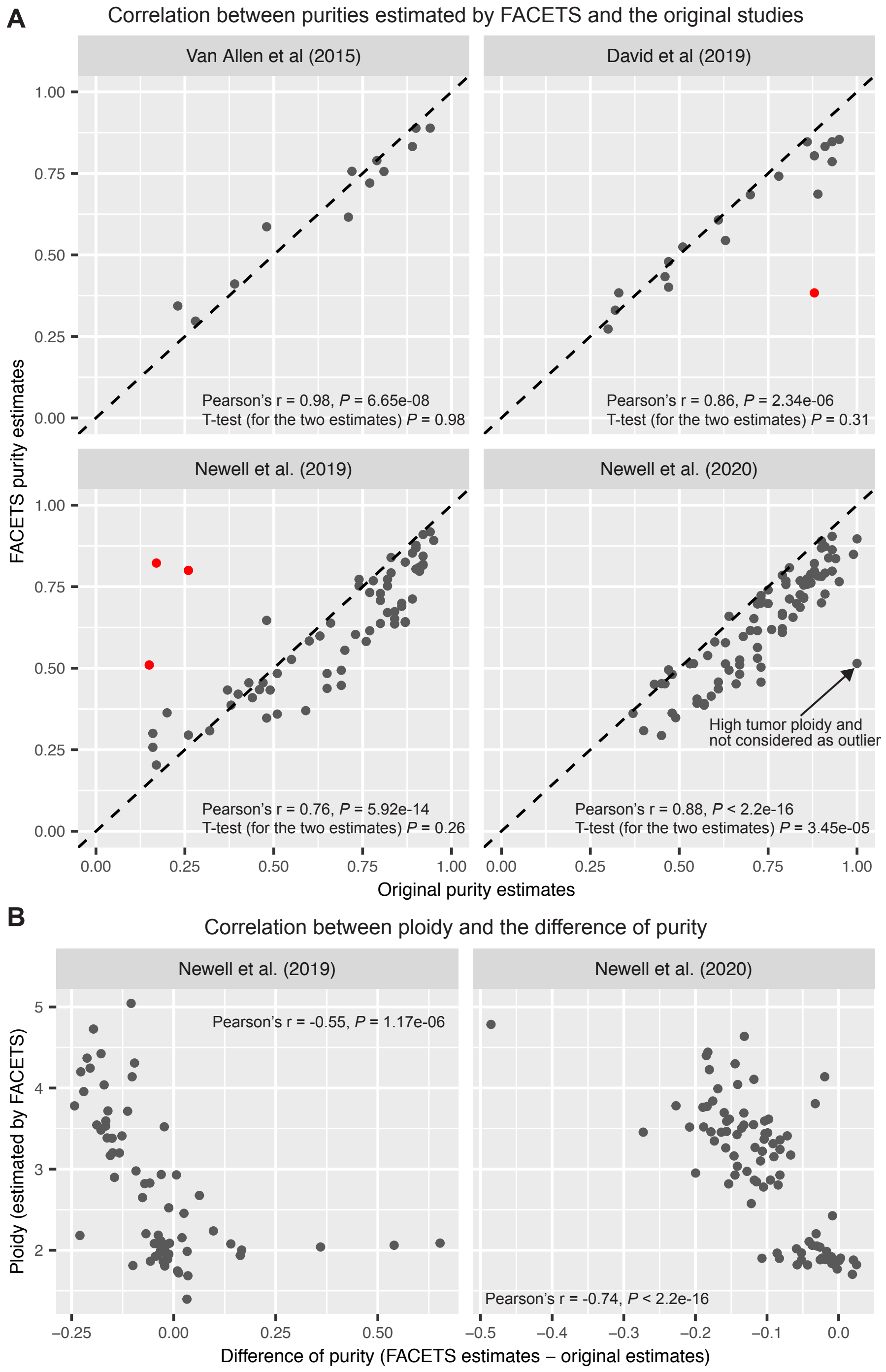

Figure S3. Tumor purities estimated through FACETS were comparable with those from the original studies with few exceptions (red dots). For the Newell et al. studies we observed larger discrepancies between our and the original estimates for hyperdiploid samples compared to near diploid samples, indicating that the original estimates probably did not account for tumor ploidy.

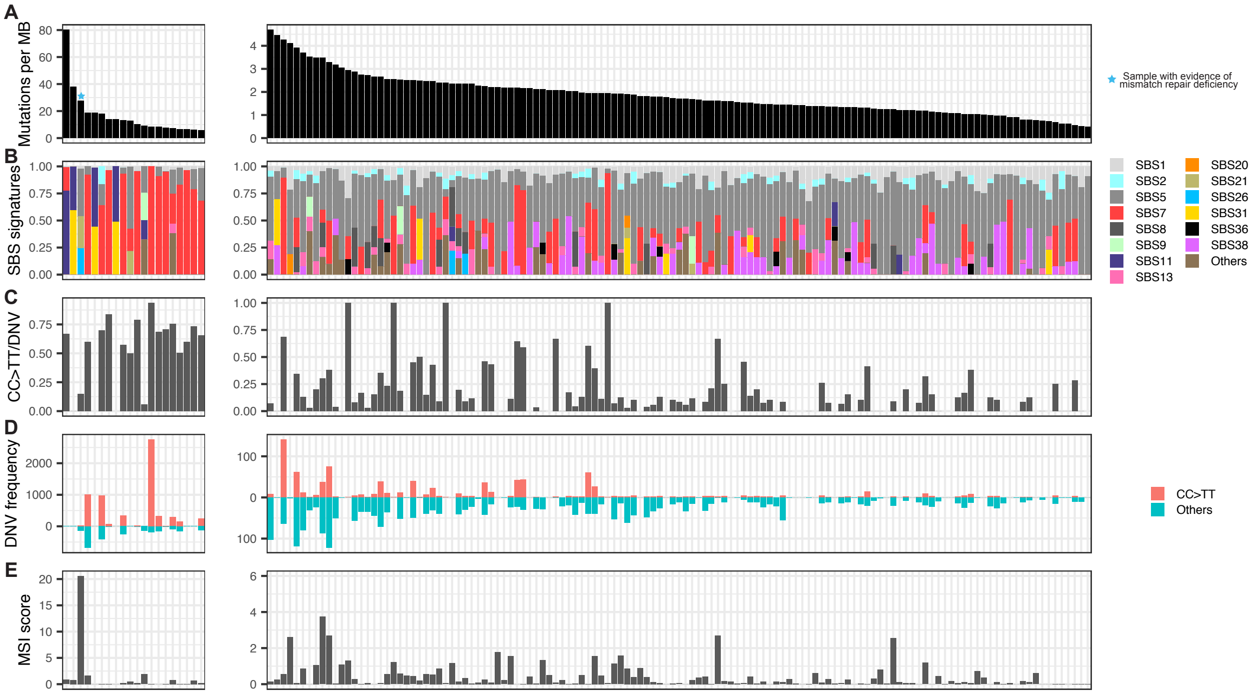

Figure S4. Features of somatic mutations in acral melanomas.

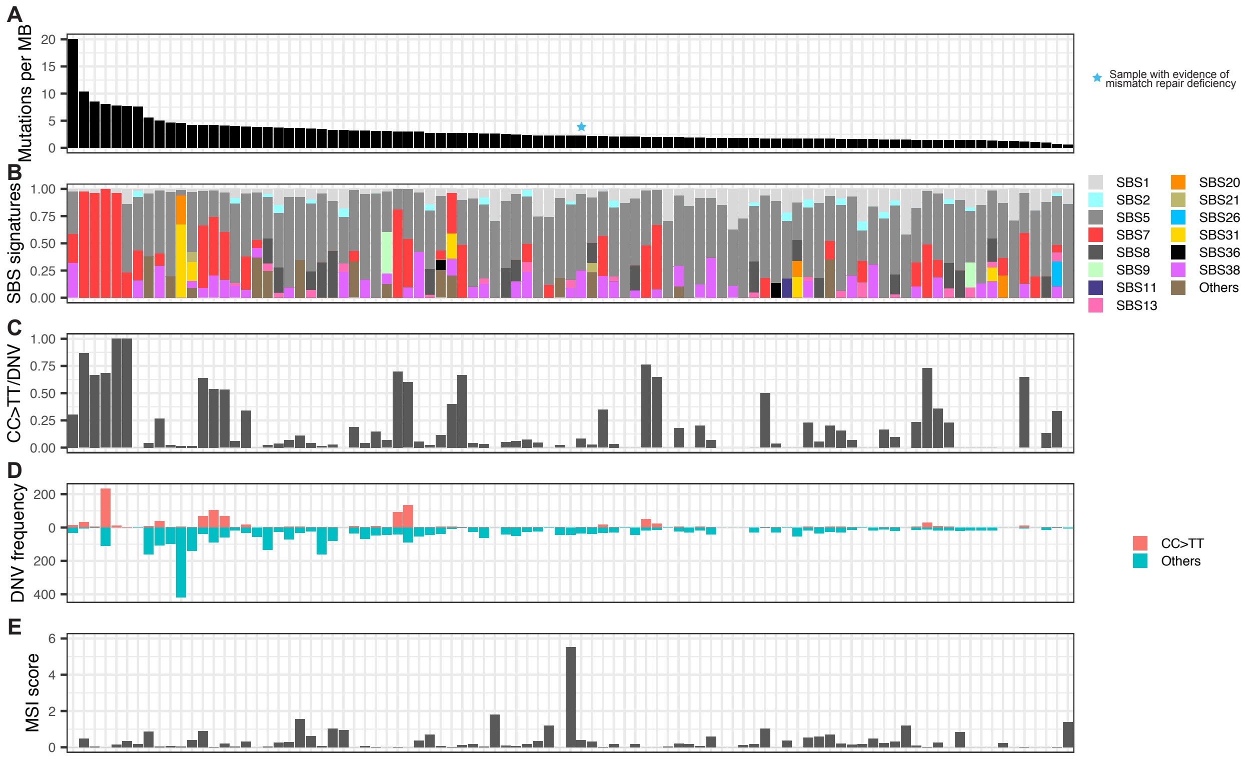

Figure S5. Features of somatic mutations in mucosal melanomas.

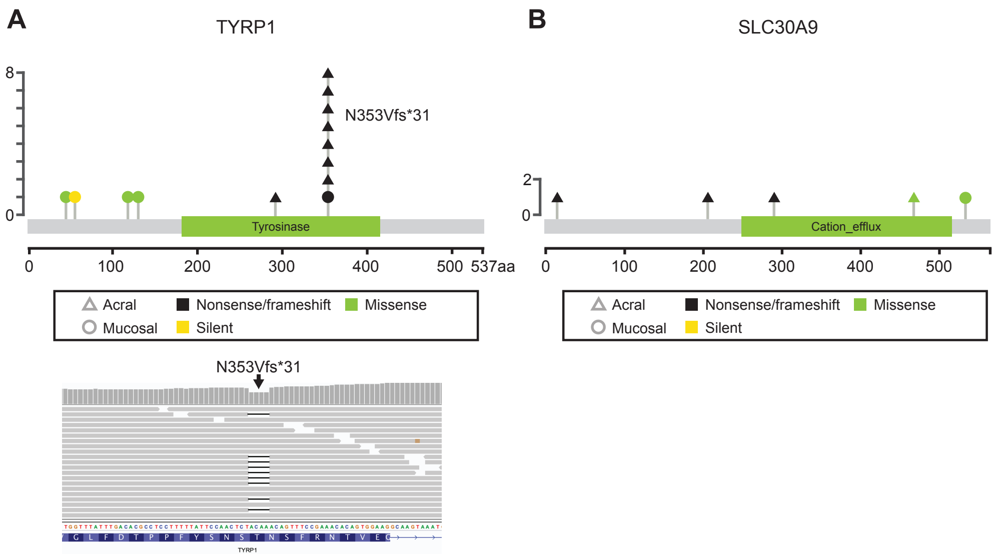

Figure S6. Lollipop plots showing somatic non-synonymous mutations on *TYRP1* and *SLC30A9*.

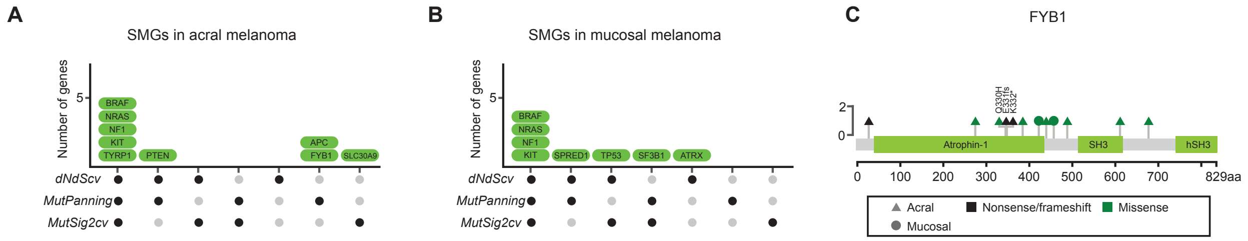

Figure S7. Significantly mutated gene (SMGs) in acral and mucosal melanomas.

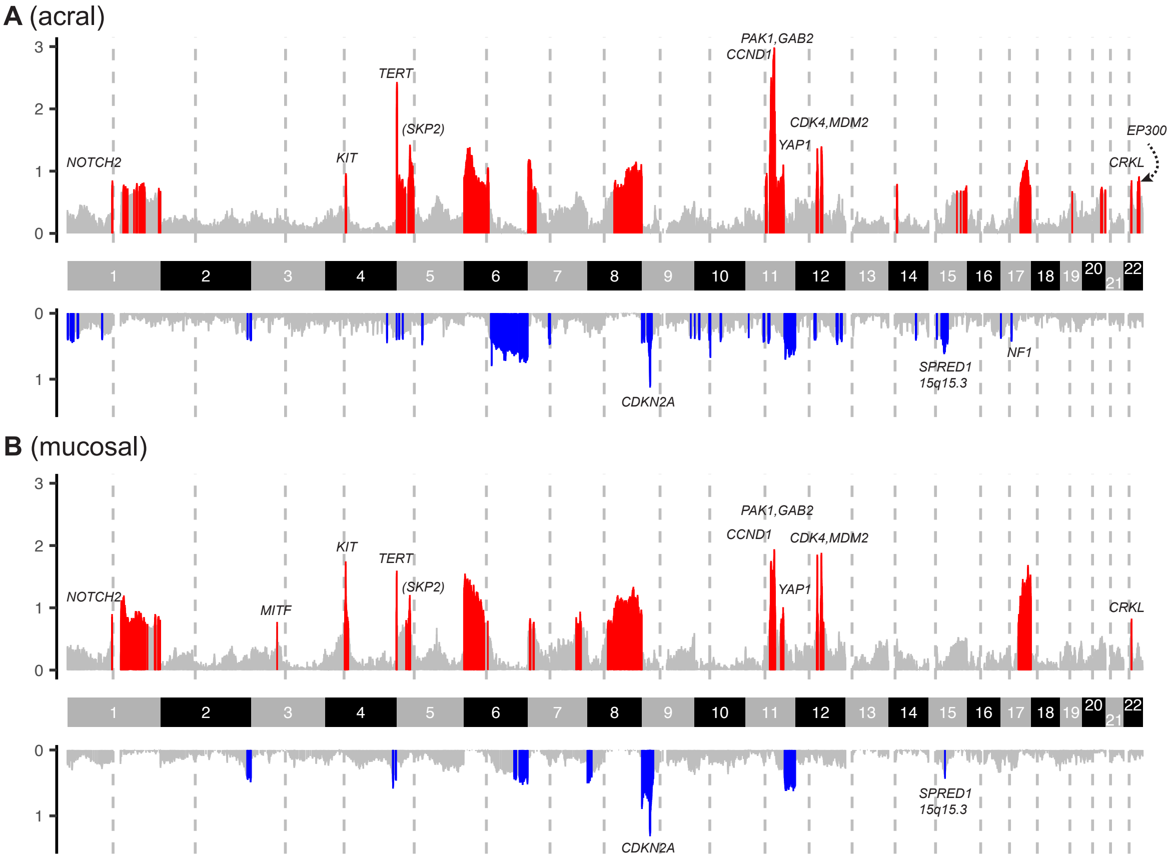

Figure S8. The GISTIC scores calculated from acral melanoma and mucosal melanoma samples.

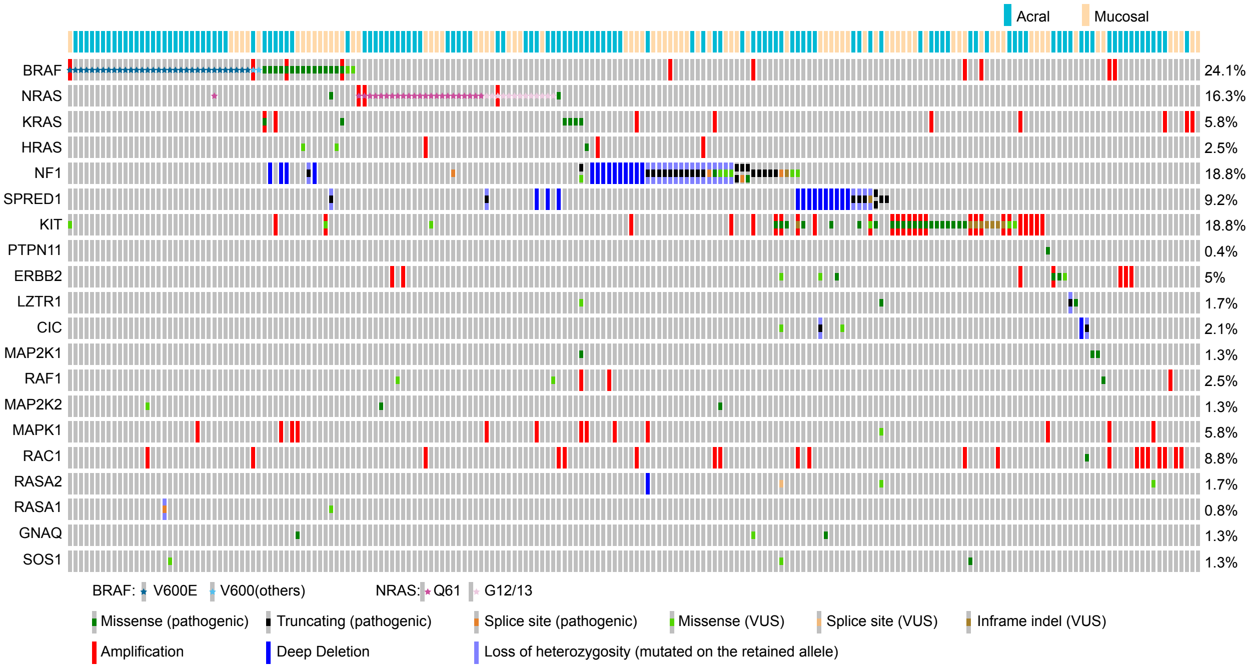

Figure S9. Oncoprint plots showing the alterations of genes that are involved in the MAPK signaling pathway.

The whole chromosome 17 (*NF1* bins marked in red)

The region surrounding NF1 (*NF1* bins marked in red)

Summary

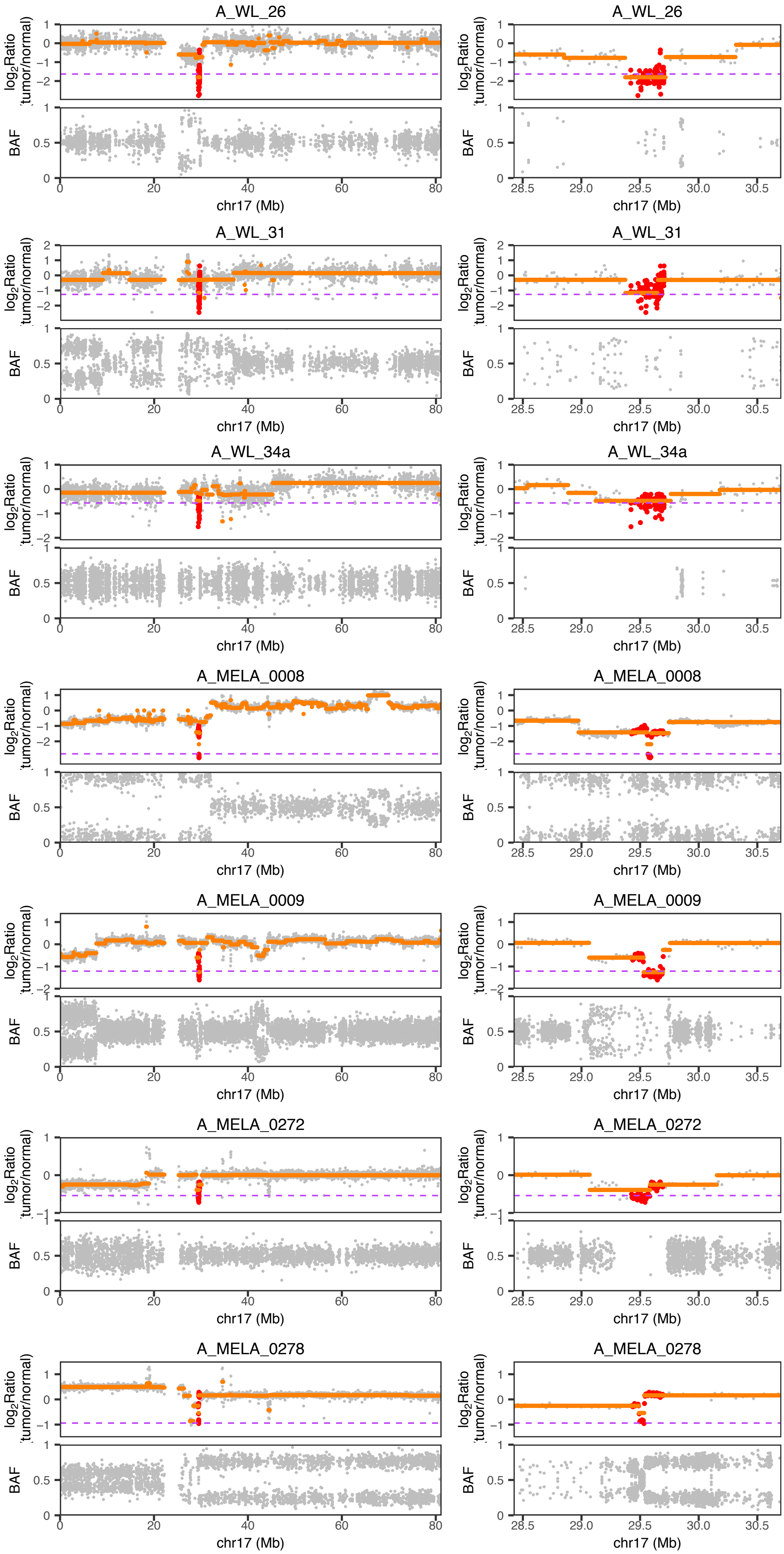

Purity = 0.68; Ploidy = 1.97

- Homozygously deleted region: the entire NF1 gene (chr17:29374144-29719037).
- The expected $\log_2$ Ratio of homozygously deleted region is -1.63 (dashed line) and expected BAF is ~0.5, in line with the observation.

Purity = 0.41; Ploidy = 4.02

- Homozygously deleted region: part of NF1 (chr17:29374927-29647538, or exons 1-35 of NM_000267).
- The expected $\log_2$ Ratio of homozygously deleted region is -1.26 (dashed line) and expected BAF is ~0.5, in line with the observation.

Purity = 0.21; Ploidy = 3.65

- Homozygously deleted region: the entire NF1 gene (chr17:29374144-29761258).
- For homozygously deleted segments, the expected $\log_2$ Ratio = -0.57 (dashed line) , in line with the observation (sample purity low and the homozygously deleted region has insuffi-

Purity = 0.73; Ploidy = 4.44

- Homozygously deleted region: part of NF1 (chr17:29565061-29595065, or exons 30-35 of NM_000267).
- The expected $\log_2$ Ratio of homozygously deleted region is -2.81 (dashed line), in line with the obsevation (homozygously deleted region has insufficient germline heterozygous SNPs).

Purity = 0.44; Ploidy = 3.35

- Homozygously deleted region: part of NF1 (chr17:29530055-29695081, or exons 12-56 of NM_000267).
- The expected $\log_2$ Ratio of homozygously deleted region is -1.21 (dashed line) and expected BAF is ~0.5, in line with the obsevation.

Purity = 0.31; Ploidy = 2

- Homozygously deleted region: part of NF1 (chr17:29335025-29585064, or exons 1-30 of NM_000267).
- The expected $\log_2$ Ratio of homozygously deleted region is -0.54 (dashed line) , in line with the obsevation (homozygously deleted region has insufficient germline heterozygous SNPs).

Purity = 0.35; Ploidy = 3.43

- Homozygously deleted region: part of NF1 (chr17:29495050-29535056, or exons 5-12 of NM_000267).
- The expected $\log_2$ Ratio of homozygously deleted region is -0.94 (dashed line) and expected BAF is ~0.5, in line with the obsevation.

Figure S10. Copy number profiles showing homozygous deletion of NF1 (page1).

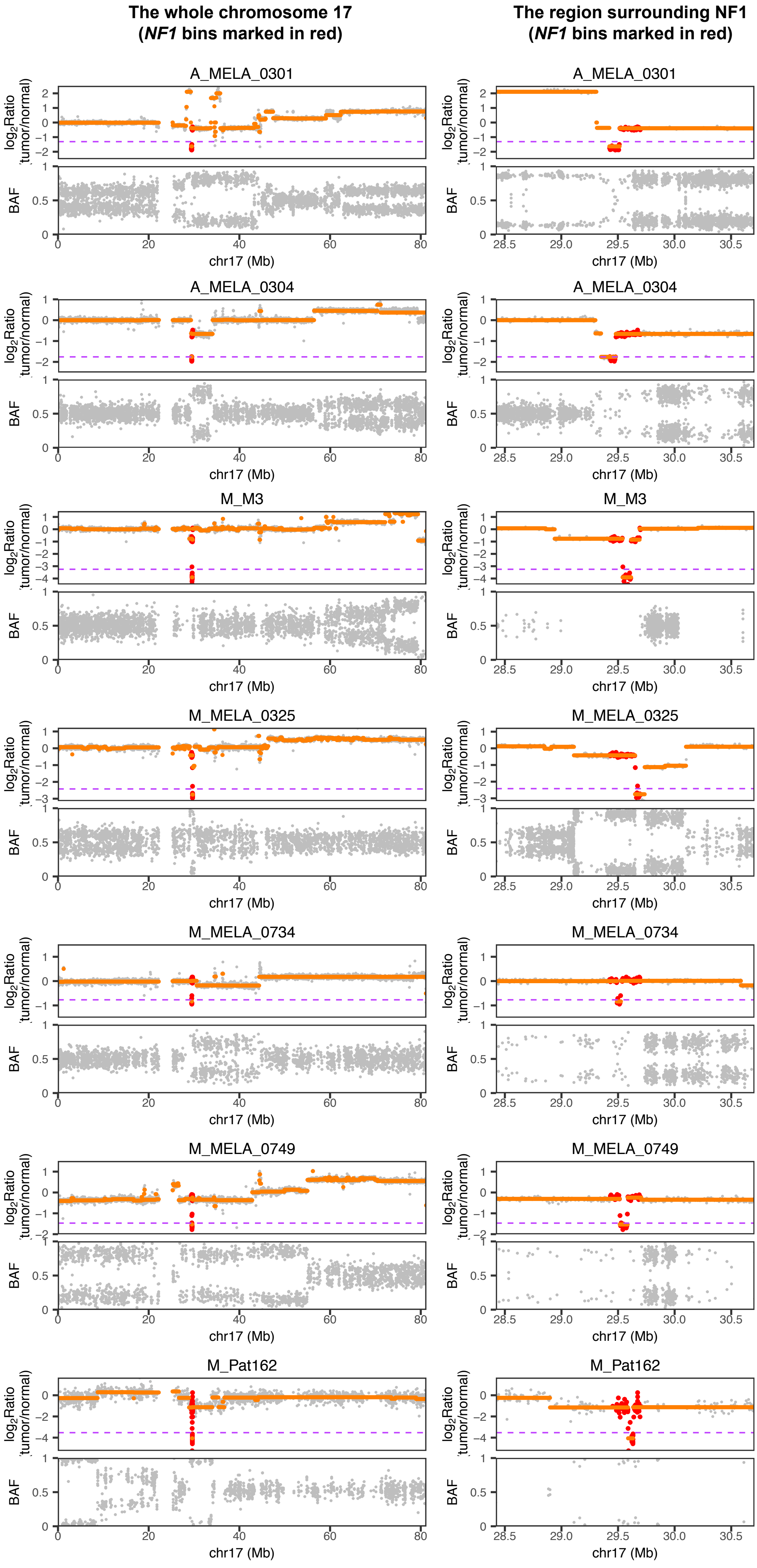

Summary

Purity = 0.46; Ploidy = 3.46

- Homozygously deleted region: part of NF1 (chr17:29425039-29515053, or exons 1-8 of NM_000267).
- The expected $\log_2$ Ratio of homozygously deleted region is -1.31 (dashed line) and expected BAF is ~0.5, in line with the obsevation.

Purity = 0.7; Ploidy = 2.05

- Homozygously deleted region: part of NF1 (chr17:29350028-29480048, or promoter, exons 1, and part of intron 1 of NM_000267).
- The expected $\log_2$ Ratio of homozygously deleted region is -1.76 (dashed line) and expected BAF is ~0.5, in line with the obsevation.

Purity = 0.88; Ploidy = 2.32

- Homozygously deleted region: part of NF1 (chr17:29540057-29615068, or exons 13-35 of NM_000267).
- The expected $\log_2$ Ratio of homozygously deleted region is -3.25 (dashed line), in line with the observation (there is insufficient germline heterozygous SNP at the homozygouosly deleted region).

Purity = 0.7; Ploidy = 3.72

- Homozygously deleted region: part of NF1 (chr17:29655074-29735087, or exons 38-57 of NM_000267).
- The expected $\log_2$ Ratio of homozygously deleted region is -2.42 (dashed line), in line with the observation (there is insufficient germline heterozygous SNP at the homozygouosly deleted region).

Purity = 0.43; Ploidy = 1.86

- Homozygously deleted region: part of NF1 (chr17:29490049-29520054, or exons 3-8 of NM_000267).
- The expected $\log_2$ Ratio of homozygously deleted region is -0.77 (dashed line) and expected BAF is ~0.5, in line with the obsevation.

Purity = 0.55; Ploidy = 2.9

- Homozygously deleted region: part of NF1 (chr17:29520054-29580063, or exons 9-31 of NM_000267).
- The expected $\log_2$ Ratio of homozygously deleted region is -1.47 (dashed line) , in line with the obsevation. (homozygously deleted region has insufficient germline heterozygous SNPs).

Purity = 0.89; Ploidy = 2.6

- Homozygously deleted region: part of NF1 (chr17:29588681-29632673, or exons 33-36 of NM_000267).
- The expected $\log_2$ Ratio of homozygously deleted region is -3.53 (dashed line) , in line with the obsevation. (homozygously deleted region has insufficient germline heterozygous SNPs).

Figure S10. Copy number profiles showing homozygous deletion of NF1 (page2).

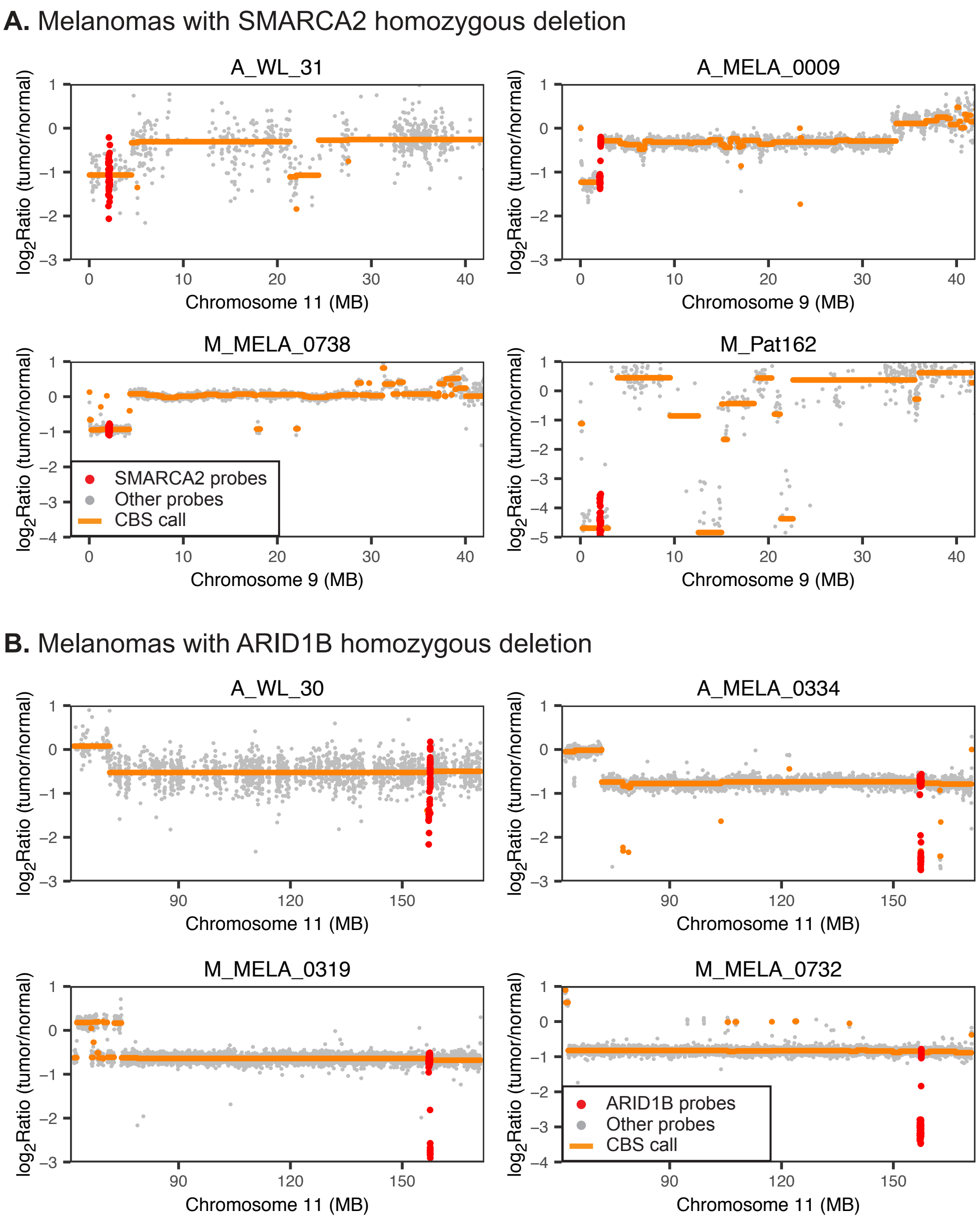

Figure S11. Copy number profiles showing homozygous deletion of *SMARCA2* and *ARID1B*.

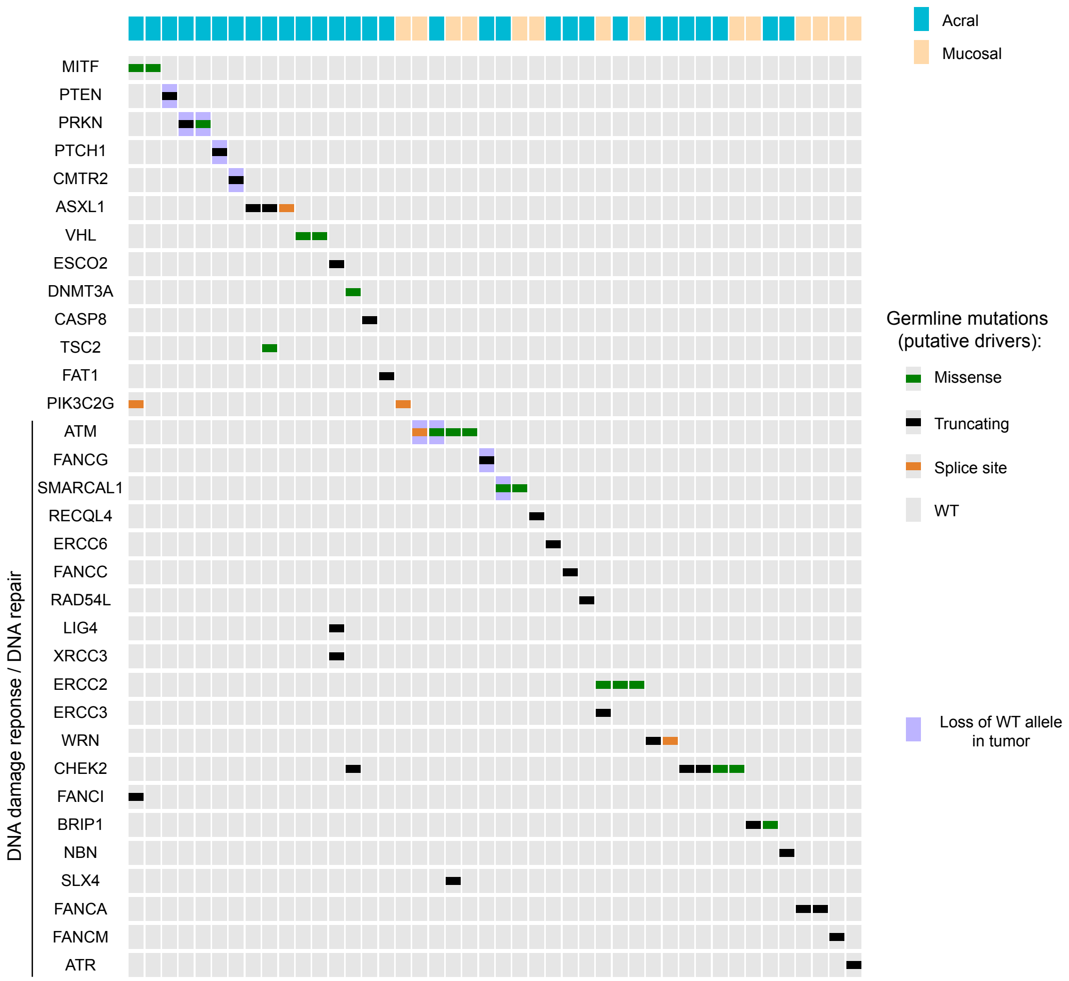

Figure S12. Tiling plot of germline mutations.

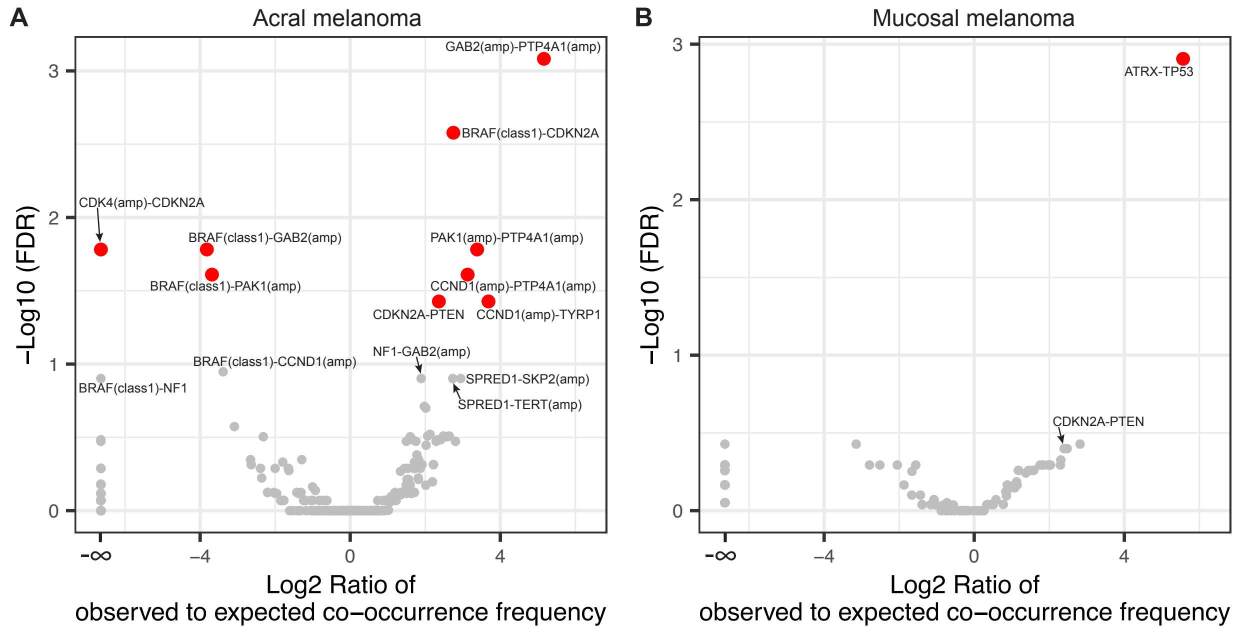

Figure S13. The mutual exclusivity and co-occurrence between pairs of driver events in acral melanoma and mucosal melanoma.

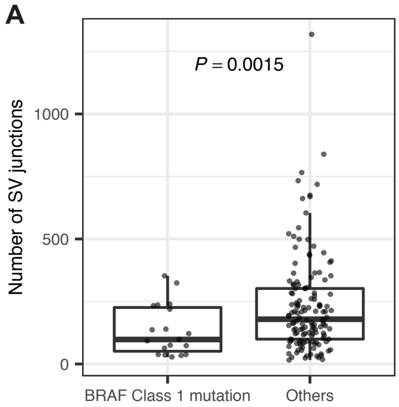

B

Multiple linear regression:
Number_of_SV ~ BRAF_Class1 + Purity + Ploidy

	Coefficient	P
Intercept	-135.03	0.09393
**BRAF_Class1(Y)**	**-96.39**	**0.03057**
Purity	263.39	0.00142
Ploidy	37.81	0.02209

Figure S14. Fewer structural variation (SV) junctions for acral and mucosal melanomas with *BRAF* class 1 mutations compared to others.

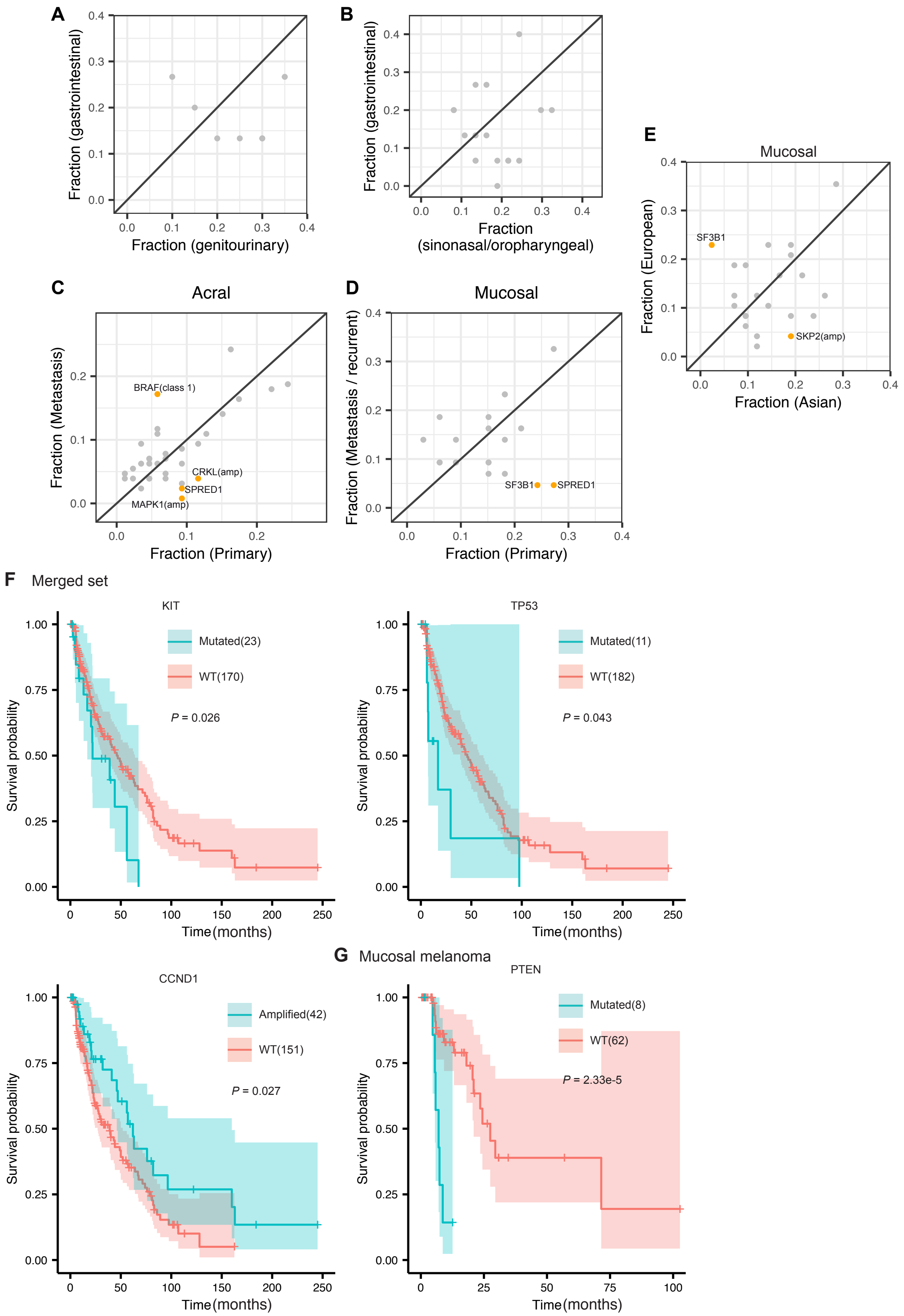

A
Fraction (gastrointestinal)
Fraction (genitourinary)
B
Fraction (gastrointestinal)
Fraction
(sinonasal/oropharyngeal)
E
Mucosal
Fraction (European)
Fraction (Asian)
SF3B1
SKP2(amp)
C
Acral
Fraction (Metastasis)
Fraction (Primary)
BRAF(class 1)
CRKL(amp)
SPRED1
MAPK1(amp)
D
Mucosal
Fraction (Metastasis / recurrent)
Fraction (Primary)
SF3B1
SPRED1
F Merged set
KIT
Mutated(23)
WT(170)
P = 0.026
Survival probability
Time(months)
TP53
Mutated(11)
WT(182)
P = 0.043
Survival probability
Time(months)
CCND1
Amplified(42)
WT(151)
P = 0.027
Survival probability
Time(months)
G Mucosal melanoma
PTEN
Mutated(8)
WT(62)
P = 2.33e-5
Survival probability
Time(months)

Figure S15. The association of genetic alterations with clinical features.
